# Patient-perspective evidence on the acceptability, usability, and everyday impact of relative motion orthoses in adult hand rehabilitation: A scoping review protocol

**DOI:** 10.1371/journal.pone.0349852

**Published:** 2026-09-11

**Authors:** Manigandan Chockalingam, Vimal Sriram, Lisa Newington, Brightlin Dhas

**Affiliations:** 1 School of Allied and Community Health, University of Galway, Galway, Ireland; 2 Institute for Health Discovery and Innovation, University of Galway, Galway, Ireland; 3 School of Public Health, Faculty of Medicine, Imperial College London, London, United Kingdom; 4 Centre for Bone and Joint Health, Blizard Institute, Queen Mary University of London, London, United Kingdom; 5 School of Health, University of Northampton, Northampton, United Kingdom; Niigata University of Health and Welfare: Niigata Iryo Fukushi Daigaku, JAPAN

## Abstract

Relative motion orthoses (RM orthoses) are finger-based orthoses used in adult hand rehabilitation to protect healing structures or optimist movement patterns while permitting active hand use. Existing reviews have mapped RM-orthosis indications, configuration, and clinical outcomes, and the most recent has begun to incorporate patient-experience findings; however, patient-perspective evidence has not yet been mapped as the primary object of a synthesis, organised against defined dimensions of acceptability, usability, and everyday impact. The objective of this scoping review is to map the extent, range, and nature of patient-perspective evidence on the acceptability, usability, and everyday impact of RM orthoses in adult hand rehabilitation. The review will follow Joanna Briggs Institute methodology for scoping reviews and be reported using the Preferred Reporting Items for Systematic Reviews and Meta-Analyses extension for Scoping Reviews. Sources will include adults aged 18 years or older who have used an RM orthosis for hand rehabilitation. Any adult hand rehabilitation context will be considered. Qualitative, quantitative, mixed-methods, case-based, survey, and grey literature sources will be included when patient-perspective data can be extracted. Ovid MEDLINE, Embase, CINAHL Complete, PsycINFO, Scopus, and Web of Science Core Collection will be searched, together with Google Scholar, ProQuest Dissertations and Theses Global, and Overton. Two reviewers will independently screen and extract data, with a third reviewer available for adjudication. Critical appraisal will not be undertaken. The protocol has been prospectively registered on the Open Science Framework (https://osf.io/dcyf8/overview). Findings will be presented in tabular, diagrammatic, and narrative form, including a summary-of-characteristics table and a concept-mapping matrix. This review will provide an evidence map to guide patient-centred orthotic practice and identify research priorities.

## Introduction

Relative motion orthoses (RM orthoses) are an established component of contemporary hand rehabilitation. A RM orthosis is a small, finger-based orthosis that positions the metacarpophalangeal joint(s) of the affected digit(s) in relatively more extension or flexion than the adjacent digits, thereby reducing the load on healing or vulnerable structures while permitting active motion of all fingers [1]. Where the injured metacarpophalangeal joint is positioned in relative extension the orthosis is termed a relative motion extension (RME) orthosis, and in relative flexion a relative motion flexion (RMF) orthosis; in the postoperative management of long extensor or flexor tendon repairs, the terms RME-*plus* and RMF-*plus* denote the addition of a wrist component and RME-*only* and RMF-*only* its absence, following Brosnan et al. [2].

The breadth of RM-orthosis clinical application has been mapped in two scoping reviews — Hirth, Howell, and O’Brien [1] and, most recently, Brosnan et al. [2]. Extensor tendon management remains the traditional indication and is supported by both general and condition-specific evidence [3–8]. Beyond it, RM orthoses are now used across a range of hand conditions, including for exercise [9], in inflammatory and degenerative conditions [10], in flexor tendon rehabilitation [3,11], for trigger finger [12–14], and in boutonnière deformity [15–17], as well as for adaptive purposes [4,10] and for sagittal band injuries, where the primary evidence is limited to earlier studies of relative-extension splinting [18,19] and no dedicated relative-motion-orthosis study has yet been reported, the available evidence being documented within the two scoping reviews [1,2].

Across these applications, the literature has become increasingly informative about indications, orthosis configuration, wear schedules, and clinical outcomes. By contrast, the evidence describing how adults experience, manage, and accommodate RM orthoses in everyday life remains scattered across diverse study designs and has only recently begun to be drawn together. The updated scoping review by Brosnan et al. [2] incorporated the emerging qualitative literature and reported patient-facing findings such as comfort, appearance, adherence, and the impact of orthosis wear on daily activities. That review, however, organises evidence by clinical indication and reports patient experience within a clinically framed synthesis: because findings are grouped by the condition treated rather than by the dimension of experience described, it does not show how a single experiential dimension — such as appearance, task compatibility, wearability, daily activities, or social participation — is represented across the conditions in which RM orthoses are used, or where it remains unexamined. A synthesis arranged by condition is built to answer questions about one condition at a time — “what is known about RM orthoses for trigger finger?”, for instance — and answers them well. It is less able to answer the reverse question this review poses: taking a single aspect of experience, such as how the orthosis looks to others or how readily it lets everyday tasks be carried out, what is known about that aspect across all the conditions RM orthoses are used for, and where is it unstudied?

The distinction matters because, as with many interventions that patients live with over time, a given aspect of experience with the same intervention — here, an RM orthosis — may carry different weight from one condition to another. Whether an orthosis is judged acceptable to wear, workable in daily use, and compatible with everyday life may be appraised differently by a person wearing it to manage trigger finger without surgery than by a person wearing it after an operation to protect a healing tendon repair, for whom the reason for wear, the duration, and what is at stake all differ. Evidence gathered in one condition therefore cannot be assumed to transfer to another, which is precisely why a cross-condition view is needed. Social participation and appearance-related concerns, for instance, appear in general patient-experience accounts of RM orthosis use [20,21] but have not, to our knowledge, been examined for indications whose evidence base is currently confined to clinical and patient-reported outcomes, such as flexor tendon rehabilitation [3,11] or trigger finger [12–14] — a cross-diagnosis pattern that a condition-by-condition arrangement cannot make visible. Indexing the map by experiential domain, and charting where evidence is concentrated or absent, is therefore intended to surface these patterns and their gaps systematically. No synthesis has yet mapped the extent, range, and nature of patient-perspective evidence on RM orthosis use as its primary object, organised by the experiential dimensions of acceptability, usability, and everyday impact.

This gap is clinically relevant. An orthosis delivers its benefit only if the patient wears and manages it in daily life, so whether it is accepted, usable, and compatible with everyday routines is not incidental to its effectiveness but a precondition of it. The value of an orthotic intervention is therefore not determined only by its biomechanical rationale or impairment-focused outcomes, but also by whether the orthosis is acceptable to wear, practical to manage, and compatible with everyday roles and routines. These experiential dimensions are not new to the field: in the broader hand and upper-limb orthosis literature, orthosis use has been linked to comfort, satisfaction, adherence-related experiences, and the practical demands of daily wear [22–25]. These factors shape whether an orthosis is tolerated, adhered to, and integrated into daily life.

For the purposes of this review, three interrelated but analytically distinct domains have been selected to organise the patient-perspective evidence: acceptability, informed by Sekhon, Cartwright, and Francis [26], which refers to the patient’s evaluative appraisal of whether the orthosis is agreeable, tolerable, and worthwhile; usability, which refers to the practical workability of the orthosis in being donned, managed, and incorporated into routines and task demands; and everyday impact, broadly informed by the International Classification of Functioning, Disability and Health (ICF) activities and participation framework, which refers to the lived consequences of orthosis wear for occupations, roles, routines, and participation beyond the clinical setting [27]. Operational definitions and sub-domains for each are provided in the inclusion criteria. Although analytically distinct, these domains may overlap in practice; findings that span them will be coded to each applicable domain (multi-domain coding). Barriers and facilitators that influence any of these domains will be recorded separately as a cross-cutting category. Together, these domains reflect a person-centred perspective on living with, managing, and accommodating an RM orthosis, and are used as analytic organisers rather than to filter or constrain eligible evidence. Studies that report only clinician or caregiver perspectives fall outside the scope of this review. Such perspectives are important and are addressed in separate literature; combining them with patient-perspective evidence in a single map would dilute the patient-centred focus that defines this review. Where therapist- or clinician-only evidence exists alongside a patient-perspective gap, this contrast will be noted in the analysis of gaps.

Understanding how patients experience RM orthoses is also important because orthosis design decisions are increasingly being guided by principles of shared decision-making and self-management [4,10]. Recent literature has emphasised the value of partnering with patients to select and tailor orthosis type, materials, and wearing regimen based on individual priorities, activity demands, and symptom patterns [4,10]. A comprehensive, patient-experience-indexed synthesis of this evidence would strengthen and consolidate the guidance already available from clinical expertise, primary qualitative research, and existing reviews, and would support more systematic, patient-centred orthotic decision-making — helping clinicians anticipate common challenges, address expectations, and tailor orthotic interventions to the realities of daily life.

However, it cannot be assumed that findings from the broader orthosis literature transfer directly to RM orthoses, which have a lower profile than many dynamic or forearm-based static orthoses, are frequently used during active hand function rather than at rest, and may be worn across a range of real-world contexts and diagnostic groups [1,3–14]. The experiential implications of these design and wear characteristics — for wearability, task compatibility, appearance, self-management, and participation — have not yet been systematically mapped for RM orthoses, and cannot simply be inferred from evidence generated for other orthosis types. Unlike the comparator orthoses used for these same indications — which protect the healing structure by restricting motion at the involved joint — RM orthoses protect by repositioning the affected finger’s metacarpophalangeal joint relative to its neighbours, permitting near-full active movement of the treated digit rather than preventing it. These distinguishing characteristics justify and support a synthesis focused specifically on relative motion (RM) orthoses. A broader comparative review — examining how the acceptability, usability, and everyday impact of RM orthoses compare with those of other orthoses used for the same indications — would address a related and valuable question that falls outside the scope of the present evidence map and is identified as a priority for future synthesis.

Existing evidence syntheses have addressed important RM-orthosis-related questions from a different analytic standpoint. Hirth, Howell, and O’Brien [1] mapped the clinical applications and evidence base for RM orthoses across hand conditions, and Brosnan et al. [2] updated that review, incorporating the qualitative literature that has since emerged. Newington, Ross, and Howell [11] reviewed relative motion flexion orthoses with emphasis on protocols and functional outcomes. Shaw and colleagues [3] extended systematic review coverage to extensor and flexor tendon repairs, again focusing on clinical effectiveness. As noted above, the present review builds on these syntheses rather than duplicating them, and does so through three distinct analytic moves. First, it re-constitutes the evidence: where prior syntheses are organised by clinical indication, orthosis-specific patient-reported findings embedded within intervention studies — an orthosis-satisfaction rating, for example — are reported there as secondary outcomes of a clinical trial, whereas the present review extracts them and treats them as patient-perspective evidence in their own right. Second, it re-indexes that evidence by experiential dimension rather than by condition, charting it against the defined domains of acceptability, usability, and everyday impact, with no date limit and with coverage extended to grey literature. Third, this reindexing makes explicit a class of gap that a condition-organised synthesis cannot expose — where a given experiential dimension is well evidenced across conditions and where it remains unexamined — yielding a gap map that can direct future primary research and orthotic decision-making toward the dimensions and populations where evidence is currently absent. Broader upper-limb and hand-orthosis reviews have addressed satisfaction and adherence-related experiences [22–25] but were not RM-orthosis-specific and did not address the broader triad of acceptability, usability, and everyday impact.

Directly relevant primary literature includes qualitative studies [20,21], randomised trials [7,13,14], and case-based reports [10] that report patient-reported comfort, wearability, satisfaction, or acceptability of the orthosis. Surveys of hand therapy practice further describe how RM orthoses are prescribed and used [5,9]. The evidence identified above is therefore heterogeneous and dispersed across qualitative studies, clinical trials, case-based reports, and surveys, and is likely to extend into grey literature not captured by previous reviews. The three domains of acceptability, usability, and everyday impact together provide a framework suited to mapping it.

A scoping review is the most appropriate approach for this evidence base. Scoping reviews are particularly suited to bodies of evidence that are emerging, conceptually broad, methodologically diverse, and not yet sufficiently mature or standardised for a focused effectiveness review [28–31]. In the present topic area, the purpose is not to estimate pooled effects or judge intervention efficacy, but to determine what patient-perspective evidence exists, how that evidence has been generated, what dimensions of acceptability, usability, and everyday impact have been addressed, and where important gaps remain.

A preliminary search of Ovid MEDLINE, the Cochrane Database of Systematic Reviews, PROSPERO, the Joanna Briggs Institute (JBI) Evidence Synthesis journal, and the Open Science Framework (OSF) was conducted on 27 March 2026. Relevant syntheses were identified [1,3,11,22–25]. However, no scoping review, systematic review, or registered protocol mapping patient-perspective evidence on the acceptability, usability, and everyday impact of RM orthoses in adult hand rehabilitation was retrieved.

The objective of this scoping review is to map the extent, range, and nature of patient-perspective evidence on the acceptability, usability, and everyday impact of RM orthoses in adult hand rehabilitation, including how this evidence has been generated, which populations and contexts have been studied, and where important gaps remain.

### Review question and sub-questions

This scoping review will address the following primary question:

- What is the extent, range, and nature of patient-perspective evidence on the acceptability, usability, and everyday impact of relative motion orthoses in adult hand rehabilitation?

Three sub-questions will guide the analysis and presentation of findings:

- In which adult populations, diagnoses, settings, and geographical contexts has this evidence been generated, and through which study designs, source types, and data-collection methods?
- Which dimensions of acceptability, usability, and everyday impact have been examined, and how have these been defined or operationalised?
- What gaps are apparent across populations, contexts, methods, and conceptual domains?

Separating the eligibility-level question from the analytical sub-questions ensures that screening decisions are made against a single primary question, while the sub-questions guide descriptive mapping of the included evidence.

## Materials and methods

The scoping review will be conducted in accordance with JBI methodology for scoping reviews [29–31]. The protocol has been registered prospectively on the Open Science Framework prior to formal screening (OSF registration identifier: https://osf.io/dcyf8/overview). Any important amendments made during review conduct will be dated, described, and justified in the completed review. The review will be reported using the PRISMA extension for Scoping Reviews (PRISMA-ScR) [32]. A completed PRISMA-P checklist is provided as S1 Checklist. Ethics approval is not required for this scoping review, as it involves only the secondary analysis of published and publicly available literature and does not include primary data collection from human participants.

### Inclusion criteria

The inclusion criteria have been structured using the Population, Concept, and Context (PCC) framework recommended by JBI for scoping reviews [29]. Because the aim of this review is to map the extent, range, and nature of patient-perspective evidence on RM orthosis use, the eligibility criteria have been framed broadly enough to capture evidence that is directly qualitative, quantitatively patient-reported, or embedded within wider clinical reports. Here, extent refers to the volume of patient-perspective evidence available across the literature, range to its breadth across populations, clinical indications, geographical contexts, study designs, and conceptual domains, and nature to the types of evidence, methods of data collection, and conceptual framings used to generate this evidence.

### Population

The review will include sources reporting on adults aged 18 years and older who have used an RM orthosis as part of hand rehabilitation. This will include adults with traumatic, post-surgical, inflammatory, degenerative, neurological, and other hand conditions in which an RM orthosis has been prescribed, fabricated, trialled, or used therapeutically. Mixed-age studies will be eligible only when adult data can be identified separately or when the source clearly concerns an adult rehabilitation population.

No restrictions will be placed on sex, gender, occupation, ethnicity, socioeconomic background, geographical location, or healthcare system. Studies focused on clinicians, students, or simulated users without patient-perspective data from adult RM orthosis users will be excluded.

### Concept

The concept of interest is patient-perspective evidence relating to the acceptability, usability, and everyday impact of RM orthosis use. For the purposes of this review, patient-perspective evidence refers to any finding reported directly by the patient, elicited from the patient through qualitative or quantitative methods, or clearly attributable to the patient’s experience of living with, managing, or using the orthosis.

Informed by Sekhon, Cartwright, and Francis [26], acceptability refers to the patient’s evaluative appraisal of whether the orthosis is agreeable, tolerable, and worthwhile, encompassing constructs such as comfort, burden, satisfaction, and emotional response; this review additionally includes appearance and willingness to continue use as operationally relevant constructs in the orthosis context.

Usability refers to the practical workability of the orthosis in being donned, managed, and incorporated into routines and task demands, including donning/doffing, fit, hygiene, skin effects, wearability, and task compatibility, as well as adherence-related experiences where these are explicitly reported by patients in relation to orthosis use. Objective adherence metrics (e.g., sensor-measured wear time) without accompanying patient-reported commentary will not be treated as patient-perspective evidence.

Broadly informed by the ICF activities and participation framework [27], everyday impact refers to the lived consequences of orthosis wear for occupations, roles, routines, and participation beyond the clinical setting; this is a review-level construct operationalised to include self-care, domestic activities, work, education, leisure, sleep, social participation, relationships, and return to valued activities.

Barriers and facilitators that influence acceptability, usability, or everyday impact will also be recorded separately as a cross-cutting category, as these may operate across all three domains. Where a finding spans more than one of acceptability, usability, or everyday impact without being specifically a barrier or facilitator, it will be coded to each applicable domain (multi-domain coding) rather than as cross-cutting.

Eligible evidence may include experience, perception, appraisal, preference, burden, benefit, adaptation, recommendation, patient-reported outcomes, satisfaction, adherence-related experiences, effects on daily activities or participation, and barriers or facilitators related to orthosis use. Evidence will be eligible whether patient-perspective evidence is primary or embedded within a broader study, provided that at least one relevant patient-perspective finding can be extracted.

To improve consistency, embedded patient-perspective evidence will be eligible only when a source reports at least one clearly patient-derived finding explicitly attributable to RM orthosis use. Eligible examples include patient quotations, interview themes, free-text responses, satisfaction or comfort ratings, usability items or adherence-related experience items, or other statements describing the experience of wearing, managing, or living with the orthosis in daily life. General functional outcomes without an explicit orthosis-experience component will not be treated as patient-perspective evidence. Orthosis-specific patient-reported data — for example an orthosis-satisfaction rating, or a comfort or wearability item referring to the orthosis — are eligible as embedded patient-perspective evidence. Generic patient-reported outcome or satisfaction measures that carry no orthosis-specific referent (e.g., a stand-alone Disabilities of the Arm, Shoulder and Hand total, or a global “satisfaction with care” score) administered during an RM-orthosis intervention will not by themselves qualify a source for inclusion; an orthosis-specific item, comment, or theme is required. This criterion is grounded in construct validity: because the object of the review is patient experience of the orthosis, an instrument that attributes nothing to the orthosis is not, in itself, evidence about that experience. To maintain transparency, where an included source reports both orthosis-specific and generic patient-reported measures, the orthosis-specific data will be charted and the presence of the generic measures noted, so that the boundary of what was and was not captured is visible to readers. We acknowledge that requiring an orthosis-specific referent necessarily shapes which sources enter the map, and that patient experience is sometimes conveyed through broader measures of function, satisfaction, or participation; this is a deliberate boundary of scope rather than a judgement of study quality, and recording the presence of any excluded generic measures alongside the charted orthosis-specific data keeps that boundary transparent and open to scrutiny.

### Context

The review will consider any adult hand rehabilitation context in which an RM orthosis is used, including outpatient hand therapy, occupational therapy, physiotherapy, hand-surgery follow-up, community or home-based rehabilitation, private practice, and patient-reported everyday environments. Hand rehabilitation is construed broadly and includes post-operative RM orthosis management delivered or supervised by hand surgeons where no separate therapy input is described, provided patient-perspective data on orthosis use are available.

All relevant clinical indications and orthosis labels will be considered. Sources will be eligible irrespective of the terminology used by authors, including relative motion orthosis, relative motion splint, yoke orthosis, yoke splint, relative motion extension orthosis, relative motion flexion orthosis, exercise relative motion orthosis, immediate relative active motion (IRAM) orthosis, and immediate controlled active motion (ICAM) orthosis, as well as related terms. Eligibility will be determined by orthosis characteristics rather than label alone. Consistent with the definitions used in the previous RM-orthosis scoping reviews [1,2], sources will be included where the orthosis has the defining feature of a relative motion orthosis: the metacarpophalangeal joint(s) of the affected finger or fingers are positioned in relatively greater extension or flexion than the adjacent fingers, with the interphalangeal joints left free to mobilise. Because terminology in this literature is inconsistent, eligibility is determined by this defining characteristic rather than by the label used; sources using any of the terms listed above, or related variants, are assessed against this characteristic at screening, and the search strategy incorporates the full range of these synonyms, eponyms, and device-name variants (S1 Appendix). No geographical limits will be applied.

### Types of sources

This scoping review will consider qualitative, quantitative, and mixed-methods research, together with case reports, case series, surveys, service evaluations, theses, dissertations, conference abstracts, and other relevant grey literature sources when at least one eligible patient-perspective finding can be extracted.

Qualitative sources may include interviews, focus groups, Photovoice, diaries, free-text survey responses, and similar approaches. Quantitative sources may include patient-reported outcome measures, satisfaction ratings, comfort ratings, adherence-related experience measures, usability questions, and other structured patient-reported data. Mixed-methods sources will be eligible where qualitative and/or quantitative patient-perspective findings are reported.

Reviews, review protocols, editorials, commentaries, technical fabrication papers, clinician-only surveys, cadaveric or laboratory studies, and studies restricted to therapist-reported or biomechanical outcomes without patient-perspective data will be excluded. Conference abstracts or grey literature sources will be retained only when sufficient methodological and result details are reported to permit extraction against the data charting form, including, at a minimum, population, orthosis characteristics, method of patient-perspective data collection, and at least one extractable patient-perspective finding. Only sources available in English, or for which an accessible English translation can be obtained, will be included in the final review. By “accessible English translation” we mean a full-text English version that is either already published, or that can be produced reliably using the resources available to the review team; sources available only as an English-language abstract, without a full text that can be read or translated in full, will be recorded and reported as excluded for language. This language limit reflects the review team’s resource and translation capacity and will be considered when interpreting the geographical and contextual distribution of the evidence.

### Search strategy

The search strategy will aim to locate both published and unpublished sources. A three-step search strategy will be used. The search strategy was developed using an initial limited search of Ovid MEDLINE and CINAHL Complete to identify relevant articles, terminology, and indexing. The text words contained in the titles and abstracts of relevant articles, and the index terms used to describe the articles, were used to develop the full Ovid MEDLINE strategy. The full strategy and hit counts are provided as S1 Appendix. The search strategy, including all identified keywords and index terms, will be adapted for each included database and information source. The reference lists of all included sources of evidence and relevant reviews will be screened for additional studies. Forward citation searching of included papers will be undertaken using Web of Science and Scopus.

To maximise sensitivity, the core database strategy searches incorporate different RM orthosis terminology, including all known synonyms, eponyms, acronyms, and device-name variants. Patient-perspective terms will not be required in the search because relevant experience-related findings may be embedded within broader clinical reports and not consistently signalled in titles, abstracts, or indexing. A hand and finger concept line is not applied at the database level; all specificity is achieved through the RM orthosis terminology itself.

Studies published in any language will be screened at title and abstract level where database records permit, using English-language titles and abstracts where provided by the database and machine translation (Google Translate) where they are not; however, only sources available in English or with an accessible English translation will be included in the final review. No date limits will be applied, and searches will not be restricted by study design. This language limit will be considered when interpreting evidence gaps.

The databases to be searched will include Ovid MEDLINE, Embase, CINAHL Complete, PsycINFO, Scopus, and Web of Science Core Collection. Sources of unpublished or grey literature will include Google Scholar (searched using three text-word combinations — (1) “relative motion orthosis” OR “relative motion splint”; (2) “yoke orthosis” OR “yoke splint”; (3) (“ICAM” OR “IRAM”) AND (“hand” OR “finger”) — with the first 200 records of each, sorted by relevance, screened; searches conducted in a private browsing session with the search date recorded, and the exact strings and dates reported in the completed review), Overton, ProQuest Dissertations and Theses Global, and conference abstract collections indexed by Embase. The full database searches will be executed at the outset of operational work, immediately before formal screening begins, so that the evidence base is current at the point of study selection. Any eligible sources published since the 27 March 2026 preliminary search — including the recently published updated scoping review by Brosnan et al. [2] — will be captured at this stage, screened, and reported in the completed review.

### Source of evidence selection

Following the search, all identified citations will be collated and uploaded into EndNote (Clarivate Analytics, PA, USA) and duplicates removed. After pilot screening of a sample of approximately 50 records to clarify the application of the eligibility criteria, titles and abstracts will be screened independently by two reviewers using Rayyan (QCRI, Doha, Qatar), with blinding to co-reviewer decisions during screening [33]. Potentially relevant sources will be retrieved in full and assessed independently in Rayyan by the same two reviewers. For sources in which patient-perspective evidence is embedded within a broader clinical report, full-text eligibility will require at least one clearly patient-derived RM-orthosis-specific finding, consistent with the operational definition stated in the inclusion criteria. Reasons for exclusion of sources at full text that do not meet the inclusion criteria will be recorded and reported in the scoping review. Any disagreements that arise between the reviewers at each stage of the selection process will be resolved through discussion, or with a third reviewer acting as adjudicator. The results of the search and the study inclusion process will be reported in full in the final scoping review and presented in a PRISMA-ScR flow diagram.

### Data extraction

Data will be extracted from papers included in the scoping review by two reviewers working independently using a piloted data extraction form developed by the reviewers in Microsoft Excel, with a third reviewer available for adjudication where required. The full extraction instrument is provided as S2 Appendix. The data extracted will include specific details about the participants, concept, context, study methods, clinical indication, orthosis terminology, timing of patient-perspective data collection, and key findings relevant to the review questions.

The draft data extraction tool was preliminarily piloted on a small purposive sample of four potentially relevant exemplar sources identified during preliminary searching and chosen to span different study designs (randomised trial, case reports, qualitative interview study, and Photovoice study) to test feasibility and completeness. Piloting confirmed that all fields could be meaningfully populated across source types. Refinements resulting from the pilot include guidance notes to distinguish the ‘concept domains addressed’ field (which domains were examined) from the ‘key findings’ field (what was found within those domains), and a note permitting case-level reporting of timing where sources contain multiple cases with different data-collection timepoints. The extraction tool will be further modified as necessary during the process of extracting data from each included evidence source, and any modifications will be detailed in the scoping review. Any disagreements that arise between the reviewers will be resolved through discussion, or with a third reviewer acting as adjudicator. If appropriate, authors of papers will be contacted to request missing or additional data.

### Critical appraisal of individual sources of evidence

Critical appraisal of individual sources of evidence will not be undertaken. This decision is consistent with JBI guidance for scoping reviews, in which the purpose is to map the extent, range, and nature of available evidence rather than to determine intervention effectiveness or certainty of findings [29–31].

### Data analysis and presentation

The evidence will be presented in tabular, diagrammatic, and narrative form to provide a descriptive map of the extent, range, and nature of patient-perspective evidence, rather than to synthesise effects or interpret intervention effectiveness [34]. Results will be organised to map the evidence landscape against the three concept domains of acceptability, usability, and everyday impact, with barriers and facilitators recorded separately as a cross-cutting category where relevant. Because these domains are analytically distinct but may overlap in practice, a finding may be coded to more than one domain when the source meaning clearly spans appraisal, practical use, and lived impact (multi-domain coding). Where sufficient data are available, patient-perspective findings will also be grouped and presented by stage of orthosis use — for example early or fitting stage, adaptation stage, and longer-term or established wear — to reflect that acceptability, usability, and everyday impact may differ across the course of orthosis use. Where sources report elapsed time in weeks since injury, surgery, or first application, this will be used to assign stage, so that grouping rests on reported timing rather than labels alone. Where sources do not report the timing of patient-perspective data, this will be recorded as a characteristic of the evidence and noted as a gap. Where screening identifies that an experiential dimension for a given indication is addressed only by clinician- or therapist-reported evidence, with no extractable patient-perspective finding, this will be recorded as a patient-perspective gap in that dimension rather than incorporated as evidence, so that areas where professional but not patient accounts exist are made visible in the gap analysis. To support consistent classification, comfort is coded under acceptability where it reflects the patient’s evaluative appraisal of the orthosis and under usability where it reflects practical wearability during tasks, with multi-domain coding applied where a single finding spans both; satisfaction, wearability, and adherence-related experiences are coded analogously against the operational definitions in the inclusion criteria. Where two reviewers classify a finding differently, the finding is coded to each applicable domain and the discrepancy resolved by discussion, or by a third reviewer where needed.

The following outputs are planned:

- A PRISMA-ScR flow diagram reporting the search and study selection process, including the number of records identified, screened, excluded (with reasons), and included.
- A summary-of-characteristics table describing all included sources by citation details, study design, population, clinical indication, setting, geographical location, and orthosis terminology (Table 1).
- A concept-mapping matrix charting each included source against the three concept domains (acceptability, usability, everyday impact) and any cross-cutting barriers or facilitators, indicating whether patient-perspective evidence was primary, embedded/secondary, or incidental (Table 2).
- A cross-tabulation of experiential coverage by clinical indication, in which the three concept domains (and their sub-domains) are arrayed against the clinical indications represented in the included evidence, so that each experiential dimension can be seen as evidenced, sparsely evidenced, or unexamined for a given indication. This dimension-by-indication matrix is the review’s primary gap-mapping output: it makes explicit where a dimension of experience is well represented across conditions and where it remains unexamined, a pattern not discernible when evidence is presented source by source or condition by condition.
- A descriptive summary table or figure mapping the distribution of included sources by study design, source type, data-collection method, and timing of patient-perspective data collection.
- A summary of populations, clinical indications, geographical distribution, and equity-relevant context where reported.

**Table 1:** Summary of characteristics of included sources (draft shell).

**Author, year**	**Country**	**Design and source type**	**Clinical indication and setting**	**Population (n, age, sex/gender)**	**Orthosis label and patient-perspective data collection**

*For each included source, the final column will record the orthosis label used by the authors (e.g., relative motion extension orthosis, yoke splint, ICAM orthosis), the patient-perspective focus (primary, embedded/secondary, or incidental), and the method used to collect patient-perspective data (e.g., interview, questionnaire, Photovoice, patient-reported outcome measure).*

**Table 2:** Concept-mapping matrix: sources mapped against concept domains (draft shell).

Source	Acceptability	Usability	Everyday impact	Barriers/ facilitators	Patient-perspective focus
**Sub-domains**	Comfort; appearance; burden; satisfaction; emotional response; willingness to continue use	Donning/doffing; fit; hygiene; skin effects; wearability; task compatibility; adherence-related experiences (patient-reported only)	Self-care; domestic activities; work; education; leisure; sleep; social participation; relationships; return to valued activities	Barriers/facilitators (specify for each)	Primary; embedded/ secondary; incidental

Draft table shells for the summary-of-characteristics table (Table 1) and concept-mapping matrix (Table 2) are provided below. A narrative summary will accompany the tabulated and charted results and will describe how the results relate to the review objective and questions, including how each experiential dimension is represented across clinical indications and where cross-diagnosis gaps are apparent, alongside identified evidence gaps more broadly. Preliminary piloting identified recurring sub-domains within each concept domain that will guide consistent coding. For acceptability, these include comfort, appearance, burden, satisfaction, emotional response, and willingness to continue use; for usability, donning/doffing, fit, hygiene, skin effects, wearability, task compatibility, and adherence-related experiences; for everyday impact, self-care, domestic activities, work, education, leisure, sleep, social participation, relationships, and return to valued activities. These represent an initial coding frame rather than a saturated taxonomy; additional sub-domains identified during extraction will be added iteratively and reported in the completed review. The presentation approach may also be refined if the nature of the included evidence requires it. If the volume or nature of retrieved evidence is insufficient to address the sub-questions, this will be reported transparently as a finding in itself, consistent with the scoping review’s aim to characterise evidence gaps.

### Study status and timeline

At the time of submission, the protocol has been finalised and prospectively registered on the Open Science Framework prior to formal screening. No stages of the review have been initiated: the full database searches have not yet been executed, no records have been screened, no full texts have been retrieved, and no data extraction has been performed. Operational work will commence shortly after submission, beginning with execution of the full database searches and pilot screening. The anticipated completion dates for the key review stages are as follows: (A) record screening (title and abstract screening followed by full-text screening) is expected to be completed by 30/11/2026; (B) data extraction is expected to be completed by 31/03/2027; and (C) results are expected to be available, and the completed scoping review submitted for publication, by 31/07/2027. Any substantive amendments to the protocol will be recorded on the OSF project page (https://osf.io/dcyf8/overview) and reported transparently in the completed review. All data underlying the completed review, including the populated data-extraction spreadsheet and PRISMA-ScR flow diagram, will be made available in the OSF project on publication.

## Supporting information

S1 Checklist PRISMA-P checklist. (DOCX)

S1 Appendix Search strategy for Ovid MEDLINE. (DOCX)

S2 Appendix Data extraction instrument. (DOCX)
