# Supplementary material for: Patient-perspective evidence on the acceptability, usability, and everyday impact of relative motion orthoses in adult hand rehabilitation: A scoping review protocol
Source: PLoS One. 2026 Sep 11;21(9):e0349852. doi: 10.1371/journal.pone.0349852 (PMC13567770; doi:10.1371/journal.pone.0349852)
Supplement: Supplementary file 1 [file pone.0349852.s001.docx]

**S1 Checklist. PRISMA-P Checklist**

*Shamseer L, Moher D, Clarke M, Ghersi D, Liberati A, Petticrew M, Shekelle P, Stewart LA; PRISMA-P Group. Preferred reporting items for systematic review and meta-analysis protocols (PRISMA-P) 2015: elaboration and explanation. BMJ. 2015;349:g7647.*

Note: Although PRISMA-P was originally developed for systematic review protocols, it is also applied to scoping review protocols where PRISMA-ScR (for completed scoping reviews) is not directly applicable at protocol stage. Item numbering follows PRISMA-P 2015. Locations are given as manuscript section names.

| **Section / item #** | **Topic** | **Checklist item** | **Location in manuscript (section)** |
| --- | --- | --- | --- |
| **ADMINISTRATIVE INFORMATION** | | | |
| 1a | Title: Identification | Identify the report as a protocol of a systematic review. | Title page. Title includes “A scoping review protocol”. |
| 1b | Title: Update | If the protocol is for an update of a previous systematic review, identify as such. | Not applicable. This is an original scoping review protocol, not an update. |
| 2 | Registration | If registered, provide the name of the registry (e.g., PROSPERO) and registration number in the Abstract. | Abstract. Materials and methods (opening paragraph). The protocol is prospectively registered on the Open Science Framework (OSF): https://osf.io/dcyf8/overview. |
| 3a | Authors: Contact | Provide name, institutional affiliation, email address of all protocol authors; provide physical mailing address of corresponding author. | Title page (authors, ORCIDs, affiliations, corresponding author email). |
| 3b | Authors: Contributions | Describe contributions of protocol authors and identify the guarantor of the review. | Contributions are entered via the PLOS submission system using the CRediT taxonomy. Guarantor: MC (Manigandan Chockalingam). See title page for the author list. |
| 4 | Amendments | If the protocol represents an amendment of a previously completed or published protocol, identify as such and list changes; otherwise, state plan for documenting important protocol amendments. | Materials and methods (opening paragraph). Any important amendments made during review conduct will be dated, described, and justified in the completed review. Amendments will also be recorded on the OSF project page (see Study status and timeline). |
| 5a | Support: Sources | Indicate sources of financial or other support for the review. | Financial Disclosure statement in submission system (no specific funding). |
| 5b | Support: Sponsor | Provide name for the review funder and/or sponsor. | Not applicable — no specific funding has been secured for this review. |
| 5c | Support: Role of sponsor or funder | Describe roles of funder(s), sponsor(s), and/or institution(s), if any, in developing the protocol. | Not applicable — no funder or sponsor involvement. |
| **INTRODUCTION** | | | |
| 6 | Rationale | Describe the rationale for the review in the context of what is already known. | Introduction. Describes RM orthoses and their clinical applications, the gap in patient-perspective evidence, the acceptability/usability/everyday impact framework, why prior reviews do not address this question, and a preliminary search confirming that no existing review or registered protocol maps patient-perspective evidence on RM orthosis use as its primary object. |
| 7 | Objectives | Provide an explicit statement of the question(s) the review will address with reference to participants, interventions, comparators, and outcomes (PICO). | Review objective stated in Introduction final paragraph. Primary question and three sub-questions in Review question and sub-questions section. The PCC (Population, Concept, Context) framework is used in place of PICO, as appropriate for a scoping review; PCC elements are specified in Inclusion criteria (with sub-sections spanning). |
| **METHODS** | | | |
| 8 | Eligibility criteria | Specify the study characteristics (e.g., PICO, study design, setting, time frame) and report characteristics (e.g., years considered, language, publication status) to be used as criteria for eligibility for the review. | Inclusion criteria opening paragraph (introduces the PCC framework [ref 24]). Population sub-section (adults ≥18 years). Concept sub-section. Context sub-section. Types of sources sub-section (English only or accessible English translation). No date limits or study-design limits; see Search strategy. |
| 9 | Information sources | Describe all intended information sources (e.g., electronic databases, contact with study authors, trial registers or other grey literature sources) with planned dates of coverage. | Search strategy. Databases listed: Ovid MEDLINE, Embase, CINAHL Complete, PsycINFO, Scopus, Web of Science Core Collection. Grey literature sources: Google Scholar, Overton, ProQuest Dissertations and Theses Global, and Embase conference abstracts. Reference-list searching and forward citation searching using Web of Science and Scopus are described in the Search strategy. No date limits. |
| 10 | Search strategy | Present draft of search strategy to be used for at least one electronic database, including planned limits, such that it could be repeated. | Search strategy section describing the three-step approach and its rationale. The full MEDLINE strategy with line-by-line syntax and hit counts is provided as S2 Appendix. |
| 11a | Study records: Data management | Describe the mechanism(s) that will be used to manage records and data throughout the review. | Source of evidence selection. Records managed in EndNote for deduplication and Rayyan for screening. Data extraction will be performed using a piloted Microsoft Excel form (Data extraction). |
| 11b | Study records: Selection process | State the process that will be used for selecting studies (e.g., two independent reviewers) through each phase of the review (i.e., screening, eligibility and inclusion in meta-analysis). | Source of evidence selection. Pilot screening of approximately 50 records. Two reviewers screen titles/abstracts and full texts independently in Rayyan with blinding. Disagreements resolved by discussion or third-reviewer adjudication. A PRISMA-ScR flow diagram will report the selection process. |
| 11c | Study records: Data collection process | Describe planned method of extracting data from reports (e.g., piloting forms, done independently, in duplicate), any processes for obtaining and confirming data from investigators. | Data extraction. Dual independent extraction using a piloted Microsoft Excel form; pilot conducted on four purposive exemplar sources spanning different study designs. Authors of included papers may be contacted for missing or additional data. The full extraction instrument is provided as S3 Appendix. |
| 12 | Data items | List and define all variables for which data will be sought (e.g., PICO items, funding sources), any pre-planned data assumptions and simplifications. | Data extraction (data items described in the Data extraction section). Full variable list in S3 Appendix. Variables include citation details, study characteristics, orthosis details, participants, timing of patient-perspective data collection, focus level, data-collection methods, concept domains and sub-domains, key findings, and reviewer notes. |
| 13 | Outcomes and prioritization | List and define all outcomes for which data will be sought, including prioritization of main and additional outcomes, with rationale. | Concept sub-section of Inclusion criteria (defines acceptability, usability, and everyday impact with sub-domains). Data analysis and presentation (describes mapping against the three domains with cross-cutting barriers/facilitators). This scoping review does not evaluate intervention outcomes; instead it maps three conceptual domains plus barriers and facilitators. |
| 14 | Risk of bias in individual studies | Describe anticipated methods for assessing risk of bias of individual studies, including whether this will be done at the outcome or study level, or both; state how this information will be used in data synthesis. | Critical appraisal of individual sources of evidence. Critical appraisal will not be undertaken, consistent with JBI scoping review guidance; rationale provided. |
| 15a | Data synthesis (quantitative) | Describe criteria under which study data will be quantitatively synthesised. | Not applicable. No quantitative synthesis will be undertaken; this is a descriptive scoping review. |
| 15b | Data synthesis (methods) | If data are appropriate for quantitative synthesis, describe planned summary measures, methods of handling data and methods of combining data from studies, including any planned exploration of consistency (e.g., I², Kendall’s tau). | Not applicable. See Materials and methods → Data analysis and presentation for the descriptive mapping approach. |
| 15c | Data synthesis (additional) | Describe any proposed additional analyses (e.g., sensitivity or subgroup analyses, meta-regression). | Not applicable. No sensitivity, subgroup, or meta-regression analyses are planned. |
| 15d | Data synthesis (qualitative) | If quantitative synthesis is not appropriate, describe the type of summary planned. | Data analysis and presentation. Results will be presented in tabular (summary-of-characteristics table, Table 1; concept-mapping matrix, Table 2), diagrammatic (PRISMA-ScR flow diagram), and narrative form. Evidence will be mapped against three concept domains with pre-specified sub-domains; multi-domain coding applied where findings span domains. |
| 16 | Meta-bias(es) | Specify any planned assessment of meta-bias(es) (e.g., publication bias across studies, selective reporting within studies). | Not applicable. Formal meta-bias assessment is not conducted in scoping reviews. Grey literature will be searched to reduce publication-bias risk at the mapping level. |
| 17 | Confidence in cumulative evidence | Describe how the strength of the body of evidence will be assessed (e.g., GRADE). | Not applicable. Confidence assessment (e.g., GRADE) is not conducted in scoping reviews, which are designed to map rather than grade evidence. |
