# Supplementary material for: Patient-perspective evidence on the acceptability, usability, and everyday impact of relative motion orthoses in adult hand rehabilitation: A scoping review protocol
Source: PLoS One. 2026 Sep 11;21(9):e0349852. doi: 10.1371/journal.pone.0349852 (PMC13567770; doi:10.1371/journal.pone.0349852)
Supplement: Supplementary file 2 [file pone.0349852.s002.docx]

**S2 Appendix. Search strategy for Ovid MEDLINE**

The search strategy below has been developed for MEDLINE (Ovid) and will be translated for the remaining databases and information sources. A preliminary scoping search of MEDLINE, Cochrane, PROSPERO, JBI Evidence Synthesis, and OSF was conducted on 27 March 2026 to check for existing or in-progress reviews mapping patient-perspective evidence on relative motion orthoses as their primary object, as reported in the manuscript. The full MEDLINE strategy below was run on 04 April 2026 to finalise the syntax and record indicative hit counts; it represents the final version of the strategy, which will be executed in full, together with its database translations, at the start of the review.

*Abbreviations used in this appendix: RM, relative motion; RM orthosis, relative motion orthosis; RME, relative motion extension; RMF, relative motion flexion; RMES, relative motion extension splint; RMFS, relative motion flexion splint; RMEO, relative motion extension orthosis; RMFO, relative motion flexion orthosis; ICAM, immediate controlled active motion; IRAM, immediate relative active motion; mIRAM, modified immediate relative active motion; mRMO, modified relative motion orthosis.*

Ovid MEDLINE(R) ALL <1946 to 04 April 2026>

Search conducted on: 04 April 2026

Limits: None applied (no date, language, or study design limits at the database level)

| **No.** | **Search strategy** | **Hits** |
| --- | --- | --- |
| 1 | ((relativ* adj2 motion adj3 (orthos* or orthot* or splint* or device*)) or (relative adj2 motion adj2 extension adj3 (orthos* or orthot* or splint* or device* or manag*)) or (relative adj2 motion adj2 flexion adj3 (orthos* or orthot* or splint* or device* or splinting)) or (exercise adj2 relative adj2 motion adj3 (orthos* or orthot* or splint* or device*)) or “relative motion extension management” or “relative motion extension splint*” or “relative motion flexion splint*” or “relative motion orthos*” or “relative motion splint*” or “relative active motion splint*” or “relative active motion orthos*” or “relative motion extension orthos*” or “relative motion flexion orthos*”).ti,ab,kf. | 69 |
| 2 | ((“relative motion” adj3 yoke) or (“digital yoke” adj3 (orthos* or orthot* or splint* or device*)) or “yoke orthos*” or “yoke splint*” or “digital yoke orthos*” or “joint active yoke” or “finger yoke” or “ICAM yoke”).ti,ab,kf. | 6 |
| 3 | (“immediate controlled active motion” or “immediate relative active motion” or ((ICAM or IRAM or mIRAM) adj3 (yoke or “relative motion” or RMES or RMFS or RMEO or RMFO or “digital yoke” or “Merritt splint*” or “Wyndell Merritt” or “Relative Motion Orthosis” or “Relative motion extension” or “Relative Motion Flexion”))).ti,ab,kf. | 8 |
| 4 | ((Wyndel* adj2 Merritt adj3 (splint* or orthos* or orthot* or protocol* or program*)) or (Merritt adj2 (splint* or orthos* or orthot* or protocol* or program*)) or (Merritt adj3 yoke) or (“modified Merritt” adj3 (splint* or orthos* or orthot*)) or “Wyndell Merritt splint*” or “Merritt splint*”).ti,ab,kf. | 2 |
| 5 | ((“border digit” adj3 splint*) or (“sagittal band bridge” adj3 splint*) or (“SB bridge” adj3 splint*)).ti,ab,kf. | 1 |
| 6 | ((RMES or RMFS or RMEO or RMFO) adj3 (splint* or orthos* or orthot* or device* or tendon* or repair* or extensor* or flexor*)).ti,ab,kf. | 4 |
| 7 | **1 or 2 or 3 or 4 or 5 or 6** | **75** |

**Note.** The strategy searches exclusively on relative motion orthosis terminology without a separate hand or finger concept line, as specificity is achieved through the RM-orthosis terminology itself. MeSH controlled vocabulary terms were not applied because no specific indexing term exists for relative motion orthoses; available broader terms (Splints; Orthotic Devices) were piloted and found to retrieve thousands of records outside the scope of this review without improving sensitivity beyond the text-word strategy. Line 1 additionally includes exact phrase variants for the extension and flexion orthosis forms (“relative motion extension orthos*”; “relative motion flexion orthos*”) to capture papers using the formal device label without the splint descriptor. Line 3 retains the full spelled-out phrases for the ICAM and IRAM programs as its primary retrieval route, supplemented by a tightly qualified adjacency construction that searches the abbreviations ICAM, IRAM, and mIRAM only when they co-occur within three words of RM-orthosis-specific vocabulary (yoke, RMES, RMFS, RMEO, RMFO, digital yoke, Merritt splint, Wyndell Merritt); this qualification eliminates noise from the intercellular adhesion molecule and radioastronomy literatures while protecting retrieval of papers that use these abbreviations primarily in the RM-orthosis context. The modified-form abbreviations mIRAM and mRMO as standalone terms were tested separately and returned zero records in MEDLINE; all papers using these abbreviations also contain the parent phrase “relative motion” captured by Line 1 or the qualified adjacency construction in Line 3. These abbreviations were tested only in MEDLINE for this protocol; they will be re-piloted in each other database during search strategy translation, and any additional retrievals will be reported in the completed review. The term “mini splint” has been used in flexor tendon rehabilitation literature (notably the Flexor Injury Rehabilitation Splint Trial [FIRST] study protocol by Bamford and colleagues, Trials 2024;25:193) to describe an RMF-type orthosis. This term was piloted separately in MEDLINE and returned no unique records not already captured by Line 1 or the related RMF terminology; it is therefore not added as a standalone search line but is noted here for transparency.
