# Supplementary material for: Patient-perspective evidence on the acceptability, usability, and everyday impact of relative motion orthoses in adult hand rehabilitation: A scoping review protocol
Source: PLoS One. 2026 Sep 11;21(9):e0349852. doi: 10.1371/journal.pone.0349852 (PMC13567770; doi:10.1371/journal.pone.0349852)
Supplement: Supplementary file 3 [file pone.0349852.s003.docx]

**S3 Appendix. Data extraction instrument**

The extraction form below was preliminarily piloted on a purposive sample of four potentially relevant exemplar sources identified during preliminary searching, chosen to span different study designs (randomised trial, case reports, qualitative interview study, and Photovoice study). Guidance notes for selected fields are provided to support consistent coding between reviewers. Any further modifications made during review conduct will be reported in the completed scoping review. Data will be extracted and charted in Microsoft Excel.

*Abbreviations used in this appendix: RM, relative motion; RM orthosis, relative motion orthosis; RME, relative motion extension; RMF, relative motion flexion.*

| **Section** | **Items to be extracted** |
| --- | --- |
| **Citation details** | Author(s); year; title; source; DOI/URL; country; language; source type (e.g. journal article, thesis, conference abstract, grey literature). |
| **Study characteristics** | Aim; design; clinical indication; setting/context; recruitment/source. |
| **Orthosis details** | Orthosis label used by authors; review-level category (e.g. RME, RMF, exercise RM orthosis, yoke); wrist component recorded as RME-*plus*/RMF-*plus* where present and RME-*only*/RMF-*only* where absent (per Brosnan et al.), with the described wrist position (e.g. neutral, extension) where reported; whether custom-fabricated or prefabricated, where reported; wear schedule if reported; fabrication material (e.g. thermoplastic, fabric, 3D-printed) where reported. |
| **Participants** | Sample size; adult age information (mean, range, or category); sex/gender where reported; other relevant participant descriptors, including equity-relevant context where reported. |
| **Timing of patient-perspective data** | During active wear; at discontinuation; follow-up; multiple timepoints within the same study; retrospective; not stated. Elapsed time where reported: weeks since injury or surgery, and weeks since the orthosis was first applied (record as reported, including ranges; record “not stated” where absent). Guidance: where a source contains multiple cases with different data-collection timepoints, report timing at the case level. Where elapsed time is reported it will be used to assign stage of orthosis use; where it is not, any stage descriptor used by the source authors will be recorded as reported. |
| **Patient-perspective focus** | Primary focus of the source; embedded or secondary; incidental mention. Guidance: a single source may contain both primary and embedded patient-perspective data (e.g. one case with patient narrative alongside cases with clinician-reported data); code the highest applicable level and note any within-source variation. |
| **Data-collection methods** | Interview; focus group; Photovoice; survey/questionnaire; patient-reported outcome measure (named); diary; observation; free-text response; mixed methods; other (specify). |
| **Concept domains addressed** | Acceptability (sub-domains: comfort, appearance, burden, satisfaction, emotional response, willingness to continue use); usability (sub-domains: donning/doffing, fit, hygiene, skin effects, wearability, task compatibility, adherence-related experiences [patient-reported only]); everyday impact (sub-domains: self-care, domestic activities, work, education, leisure, sleep, social participation, relationships, return to valued activities); barriers/facilitators; other relevant domains. Guidance: record which domains and sub-domains were examined in the source; do not record what was found here (see Key findings). Where a finding clearly spans domains, more than one domain may be coded. Coding anchor examples: comfort is coded under acceptability where it reflects the patient’s evaluative appraisal of the orthosis (e.g. “the orthosis felt tolerable to wear”) and under usability where it reflects practical wearability during tasks (e.g. “the orthosis rubbed during gripping”); satisfaction, wearability, and adherence-related experiences are coded analogously against the operational definitions in the inclusion criteria. Inter-reviewer decision rule: where two reviewers classify the same finding differently, the finding is coded to each applicable domain (multi-domain coding) and the discrepancy resolved by discussion, or by a third reviewer where needed. |
| **Key findings** | Findings relevant to acceptability, usability, everyday impact, and barriers/facilitators; any patient quotations if qualitative. Guidance: record what was found within the domains identified above; include direction and magnitude of findings where available. |
| **Reviewer notes** | Methodological observations; data quality concerns; queries for author contact; any other notes relevant to the charting process. |
