# Supplementary material for: Patient-perspective evidence on the acceptability, usability, and everyday impact of relative motion orthoses in adult hand rehabilitation: A scoping review protocol
Source: PLoS One. 2026 Sep 11;21(9):e0349852. doi: 10.1371/journal.pone.0349852 (PMC13567770; doi:10.1371/journal.pone.0349852)
Supplement: Supplementary file 5 [file pone.0349852.s005.docx]

**Response to Reviewers**

PLOS ONE | Manuscript PONE-D-26-20165

*Patient-perspective evidence on the acceptability, usability, and everyday impact of relative motion orthoses in adult hand rehabilitation: A scoping review protocol*

We thank the Staff Editor and both reviewers for their careful and constructive assessment of our protocol. We are grateful for Reviewer #1’s positive appraisal and minor-revision recommendation, and for Reviewer #2’s detailed comments, which have materially strengthened the manuscript. The most significant development since submission is the publication of the updated scoping review by Brosnan et al. (2026), which appeared after our preliminary search; we have engaged with it fully. Because several comments from both reviewers relate to it, we set out our position once, in Section A, and refer back to it where relevant.

*For each point, we give our response and, where the change is a specific wording change, the exact revised text in a shaded “REVISED TEXT” block that can be read directly against the manuscript. Reviewer and editor comments are reproduced verbatim in italics; the short headings and the R1/R2 numbering are ours, added for navigation only. Where a comment is brief, we identify the submitted sentence it was attached to. Revisions we have made on our own initiative are headed “Additional revision by the authors.” Line references are to the submitted manuscript.*

*Note on references: Brosnan et al. (2026) was published after submission and therefore does not appear in the submitted manuscript. In the revised manuscript it is added as a new reference, integrated into the reference list in order of first appearance and cited as [2], immediately after Hirth et al. [1]; all references that previously followed Hirth et al. are shifted down by one position accordingly, and the reference list is renumbered in order of first appearance. The full citation is: Brosnan M, Hirth MJ, O’Riordan C, Morrissey A-M. Ten years on: an updated scoping review of relative motion orthoses for managing various hand conditions. J Hand Ther. 2026 (in press). doi:10.1016/j.jht.2026.02.006.*

**A. How the proposed review relates to Brosnan et al. (2026) — our central position**

This underlies Reviewer #1’s comment R1.2 and several of Reviewer #2’s comments; we state it here and cross-reference it throughout.

Brosnan et al. (2026), an update of Hirth et al. (2016), appeared after our preliminary search of 27 March 2026 and was not available at drafting. We agree it is directly relevant and have revised the manuscript to acknowledge and engage with it. We first correct our own framing. Our submitted text asserted that “there has been no comprehensive synthesis mapping the extent, range, and nature of patient-perspective evidence on RMO use across study designs, populations, and contexts” (lines 53–56). With Brosnan et al. now published, that assertion is no longer defensible as written, and we have removed it. In its place the revised introduction makes the claim we can substantiate: that no synthesis has yet mapped patient-perspective evidence on RM orthosis use as its primary object, organised by the experiential dimensions of acceptability, usability, and everyday impact. This is a more precise claim than the one we submitted, not a lesser one: it names the gap this review is designed to close, it is verifiable against both published reviews (the four axes below), and the manuscript sets out why closing it matters — an orthosis delivers its benefit only if the patient wears and manages it in daily life, so acceptability, usability, and everyday impact are preconditions of its effectiveness rather than incidental to it. Brosnan et al. appraise four studies containing qualitative data (the interpretive qualitative studies of Arslan et al. and Cole et al.; the qualitative case report of Feehan et al.; and the mixed-methods study of Mottay et al.) and report patient-facing findings including comfort, skin effects, appearance, adherence, expectations, and the impact of orthosis wear on daily activities and emotional and social life. We do not claim these experiences are undocumented, but reporting them within an indication-organised synthesis is not the same as charting them as evidence in their own right.

Our review is distinct and complementary, along four axes verifiable against the two published reviews and our own protocol:

1. Object and organisation. Brosnan et al. and Hirth et al. organise the evidence by clinical indication (which conditions RME/RMF orthoses are used for; level of evidence per condition; recommended MCPJ differential; preferred design). Brosnan et al. present their qualitative and patient-experience material within these indication categories (protective, exercise, adaptive) and draw it together narratively in their discussion. Our review makes patient-perspective evidence the sole object of the map and charts it against three pre-specified, operationally defined domains — acceptability (informed by Sekhon et al.), usability, and everyday impact (informed by the ICF) — with a concept-mapping matrix as its primary output. The difference is the mode of organisation: because Brosnan et al. index by indication and synthesise experience narratively, their account does not systematically chart how a single dimension of experience — appearance, task compatibility, wearability, sleep, or social participation — is represented across conditions, or where such evidence is absent. A map indexed by experiential domain is built to make those cross-diagnosis patterns, and their gaps, explicit and systematic rather than narrative.

2. What is extracted from shared sources. Brosnan et al. include primary intervention studies and report the patient-reported outcomes within them (for example orthosis satisfaction, reported alongside range of motion and pain) as clinical outcomes, organised by indication. Our review takes the same class of source but extracts and charts those orthosis-specific patient-reported findings — orthosis-satisfaction ratings, comfort, wearability, and adherence-related experience — as patient-perspective evidence in their own right, against the acceptability/usability/everyday-impact framework. The difference is not which sources are eligible but what is extracted from them and how it is charted: data that appear in Brosnan et al. as secondary clinical endpoints are, in our review, treated as primary units of a patient-experience map. (For accuracy: Brosnan et al. did exclude secondary source types — cross-sectional studies, narrative and systematic reviews, and clinical guidelines — per their Table 2; our point on this axis concerns the primary studies common to both reviews.)

3. Source breadth and time coverage. Brosnan et al. searched four databases (MEDLINE, Embase, PubMed, CINAHL), excluded grey literature, and applied a November 2015–August 2025 window. Hirth et al. (2016) covered the earlier literature, but the patient-facing content it captured appears there as effectiveness outcomes, orthosis-design observations, or expert opinion — not as patient-perspective evidence charted against an experiential framework — and that earlier literature was not revisited by Brosnan et al. (whose criteria excluded any publication included in the 2016 review). Our review searches six databases (Ovid MEDLINE, Embase, CINAHL Complete, PsycINFO, Scopus, Web of Science Core Collection), and the additions of PsycINFO, Scopus, and Web of Science specifically index the patient-experience and psychosocial literature central to this review; it also applies no date limit and admits grey literature and theses. Its included set and charted content are therefore expected to extend beyond, rather than replicate, Brosnan et al.

4. Analytic output. Our concept-mapping matrix codes each source as primary, embedded/secondary, or incidental against the three domains, producing an explicit map of where patient-experience evidence is concentrated and where it is absent across populations, diagnoses, methods, and conceptual domains. This gap map has a downstream value neither prior review provides: by making the shape of the evidence — and its holes — explicit, it allows future primary research to be targeted at genuinely under-evidenced domains and populations rather than duplicating well-covered ground, and it signals to clinicians where current practice necessarily rests on extrapolation beyond the available patient-perspective evidence. Neither prior review produced an equivalent artefact.

We are candid that the small existing body of dedicated qualitative RM-orthosis research — the studies by Arslan, Cole, Feehan, and Mottay — is already included in Brosnan et al., and our contribution therefore lies in the analytic lens and the breadth of additional source types rather than in identifying previously undiscovered studies. We are equally candid on the clinical rationale: the prior reviews and the primary qualitative studies already provide clinicians with substantive experiential guidance, and we do not claim to supply knowledge those sources lack. What the present review adds is structure — a patient-experience-indexed map with an explicit gap analysis — that allows researchers to locate the experiential evidence relevant to a given dimension of use and to target future study at under-evidenced domains, and allows clinicians to recognise where that evidence is thin or absent and current practice therefore rests on extrapolation. This directly addresses the reviewers’ shared request to show how the review builds on, rather than duplicates, the existing syntheses.

*We note PLOS ONE’s guidance that there is no obligation to cite reviewer-recommended works; we cite and engage Brosnan et al. because it is genuinely central to the rationale.*

**B. Response to Reviewer #1**

We thank Reviewer #1 for the positive appraisal and for three suggestions, all adopted.

**R1.1 (timing of patient-perspective outcomes).** *The protocol appropriately identifies the timing of patient-perspective data collection as a data extraction item. However, patient experiences such as comfort, satisfaction, usability, adherence-related experiences, and everyday impact may differ substantially according to the stage of orthosis use (e.g., immediately after fitting, during the adaptation period, or after long-term use). It would be helpful if the authors could clarify how timing-related information will be synthesized and presented in the final review. For example, will findings be grouped according to stages of orthosis use where sufficient data are available? Such clarification may enhance the interpretability of the resulting evidence map.*

**Response.**

We agree. The protocol already records timing of patient-perspective data collection as a charting item (Data extraction, lines 322–323). We have added to the Data analysis and presentation section a sentence specifying that, where data allow, findings will be grouped and presented by stage of orthosis use, and that where sources do not permit staging this will be recorded as a characteristic of the evidence and noted as a gap. Where sources report elapsed time (weeks since injury or surgery, or since the orthosis was first applied), this will be used to assign stage, so that grouping rests on reported timing rather than on stage labels alone; the data extraction form (S3 Appendix) has been updated accordingly to record elapsed time where available.

*To be added to the Data analysis and presentation section (after the sentence ending “…(multi-domain coding).” at line 352):*

**REVISED TEXT**
Where sufficient data are available, patient-perspective findings will also be grouped and presented by stage of orthosis use — for example early or fitting stage, adaptation stage, and longer-term or established wear — to reflect that acceptability, usability, and everyday impact may differ across the course of orthosis use. Where sources report elapsed time in weeks since injury, surgery, or first application, this will be used to assign stage, so that grouping rests on reported timing rather than labels alone. Where sources do not report the timing of patient-perspective data, this will be recorded as a characteristic of the evidence and noted as a gap.

**Change made:** Data analysis and presentation — added the stage-of-use grouping sentence above. S3 Appendix (data extraction form), Timing field — added capture of elapsed time where reported (weeks since injury or surgery, and weeks since the orthosis was first applied), and the rule that stage of orthosis use is assigned from elapsed time where reported, and from the source authors’ own stage descriptor where it is not. (Additive; does not alter registered scope.)

**R1.2 (further justification for an RM-orthosis-specific review).** *The rationale for an RMO-specific review is generally convincing. However, given that several recent qualitative and patient-experience studies have emerged in this area, the authors may wish to further highlight how the growing patient-perspective evidence base has created the need for a dedicated synthesis. This may help further distinguish the current review from previous RMO reviews that primarily focused on clinical outcomes and orthosis application.*

**Response.**

We have strengthened this rationale, drawing on Section A. The revised “Existing evidence syntheses” paragraph — shown in full in the REVISED TEXT block under R2 detailed — include Brosnan in the existing-syntheses paragraph, below — now notes the emergence of dedicated qualitative RM-orthosis research since 2016 and frames the present review as the patient-experience-indexed synthesis this growing but dispersed evidence now warrants — complementary to, and building on, Hirth et al. and Brosnan et al.

**Change made:** Introduction — addressed within the revised “Existing evidence syntheses” paragraph, which now names Brosnan et al. and the emergence of qualitative RM research. No separate REVISED TEXT block is shown here, to avoid reproducing the same paragraph twice; it appears in full at the block named in the response above.

**R1.3 (transparency of coding across domains).** *The conceptual framework of acceptability, usability, and everyday impact is a notable strength of the protocol. However, several patient-reported constructs (e.g., comfort, satisfaction, wearability, and adherence-related experiences) may reasonably overlap across domains. Although the protocol indicates that multi-domain coding will be used when appropriate, providing additional coding examples or decision rules within the extraction framework may further improve transparency and reproducibility. This may be particularly useful when different reviewers are required to classify findings that could plausibly fit more than one conceptual category.*

**Response.**

We agree. The protocol already specifies multi-domain coding (lines 349–352). We have added anchor coding guidance and an explicit inter-reviewer decision rule to the Data analysis and presentation section, and fuller anchor examples — including verbatim coding examples — to the extraction instrument (S3 Appendix).

*To be added to the Data analysis and presentation section, following the multi-domain-coding provision (line 352) and the stage-of-use sentence added under R1.1:*

**REVISED TEXT**
To support consistent classification, comfort is coded under acceptability where it reflects the patient’s evaluative appraisal of the orthosis and under usability where it reflects practical wearability during tasks, with multi-domain coding applied where a single finding spans both; satisfaction, wearability, and adherence-related experiences are coded analogously against the operational definitions in the inclusion criteria. Where two reviewers classify a finding differently, the finding is coded to each applicable domain and the discrepancy resolved by discussion, or by a third reviewer where needed.

**Change made:** Data analysis and presentation — added the coding rule above. S3 Appendix — added fuller anchor examples, with verbatim coding examples, and the same decision rule. (Additive; strengthens the existing multi-domain-coding provision.)

**C. Response to Reviewer #2**

**R2 General (a).** *The recent scoping review on relative motion orthoses should be incorporated into this study protocol. The authors should clearly acknowledge this review and explain how the proposed review builds on, rather than duplicates, the existing evidence base.*

**Response.**

We agree, and have engaged Brosnan et al. throughout the revised introduction; our full position is in Section A. In brief, we have removed the superseded novelty claim, added Brosnan et al. as the most recent and directly relevant synthesis, and stated explicitly how our review builds on it — by making patient-perspective evidence the organising object, charting it against a defined framework, extracting patient-reported outcomes as experiential evidence, reaching grey literature and the pre-2016 evidence Brosnan et al. did not cover, and producing an explicit gap map. The specific wording changes are shown in the REVISED TEXT blocks under R2 detailed — structured overview of clinical applications; R2 detailed — “no comprehensive synthesis” assertion; and R2 detailed — include Brosnan in the existing-syntheses paragraph, all below.

**Change made:** Introduction — Brosnan et al. added and engaged. No separate REVISED TEXT block is shown here: the change is carried by the three introduction passages named in the response above, each reproduced with its own block below. Search strategy — the corresponding change is shown under R2 detailed — update the search; acknowledge Brosnan since published, below.

**R2 General (b).** *In my opinion, the proposed review question is overly narrow and may not address the questions that are most relevant to clinicians. Relative motion orthoses are typically one of several orthotic options used for the same diagnosis or therapeutic purpose, yet comparator orthoses are excluded without sufficient justification. For example, trigger finger may involve MCPJ-blocking orthoses; extensor tendon rehabilitation may involve wrist-hand-finger orthoses (WHFOs) with the IP joints free, relative motion approaches, or Norwich Regime; and flexor tendon rehabilitation may involve dorsal blocking, Manchester Short, or other protocols etc. A broader comparative synthesis would likely provide greater clinical value by allowing therapists to compare the acceptability, usability, and everyday impact of the orthotic options commonly considered for the same clinical presentation. If the authors elect to retain the current review scope, the rationale for excluding comparator orthoses should be strengthened.*

**Response.**

We have retained the RM-orthosis-specific scope, which the reviewer explicitly invites as an alternative to broadening, and strengthened its justification. A single-concept scope is common and legitimate in scoping reviews. We have also added a sentence to the introduction identifying the biomechanical distinction that separates RM orthoses from the comparators the reviewer lists: those comparators (MCPJ-blocking, WHFO or Norwich, and dorsal-blocking or Manchester Short) protect the repair by restricting motion at the involved joint, whereas the RM orthosis protects by repositioning the affected MCP joint relative to its neighbours so that near-full active movement of the treated digit is permitted rather than prevented. Because the wearing experience of an orthosis used during active hand function differs from that of one that blocks the target joint, the patient-perspective evidence for the RM approach is not interchangeable with that for its comparators, and pooling them would obscure exactly the distinctions this review is designed to map. We would add that centring the patient’s own experience is not a narrowing of the evidence base but the completion of an axis it has so far left largely implicit: the existing syntheses map RM orthoses chiefly by indication, configuration, and clinical outcome, whereas how patients find them to wear, use, and live with has not been drawn together. Mapping that dimension is consistent with the client-centred orientation of contemporary hand rehabilitation, in which the person’s own account of an intervention informs how it is selected and used. On this reading the review’s focus is not narrower than a comparative one but complementary to it — it supplies the patient-perspective layer on which an informed comparison of orthotic options would itself depend. We have revised the closing sentence of the transfer-of-evidence paragraph both to soften “necessary rather than optional” (see the dedicated comment below) and to position a comparative review explicitly as valuable future work.

*Replaces the final sentence of the paragraph at lines 95–96 (“This makes RMO-specific mapping… necessary rather than optional.”):*

**REVISED TEXT
Unlike the comparator orthoses used for these same indications — which protect the healing structure by restricting motion at the involved joint — RM orthoses protect by repositioning the affected finger’s metacarpophalangeal joint relative to its neighbours, permitting near-full active movement of the treated digit rather than preventing it. These distinguishing characteristics justify and support a synthesis focused specifically on relative motion (RM) orthoses. A broader comparative review — examining how the acceptability, usability, and everyday impact of RM orthoses compare with those of other orthoses used for the same indications — would address a related and valuable question that falls outside the scope of the present evidence map and is identified as a priority for future synthesis.**

**Change made:** Introduction — replaced the “necessary rather than optional” sentence with the above; scope unchanged (consistent with OSF registration).

**Additional revision by the authors — scope boundary (in support of R2 General (b)).** *To make the scope boundary explicit and further address the “overly narrow” concern, we have added a sentence stating why clinician- and carer-only perspectives fall outside this review.*

**Response.**

We have added a sentence to the introduction making explicit that studies reporting only clinician or caregiver perspectives fall outside the scope, and why. This clarifies the deliberate patient-perspective boundary and complements the strengthened scope rationale above.

*To be added to the introduction, at the end of the paragraph establishing the patient-perspective focus (after the sentence ending “…filter or constrain eligible evidence.” at line 79):*

**REVISED TEXT**
Studies that report only clinician or caregiver perspectives fall outside the scope of this review. Such perspectives are important and are addressed in separate literature; combining them with patient-perspective evidence in a single map would dilute the patient-centred focus that defines this review. Where therapist- or clinician-only evidence exists alongside a patient-perspective gap, this contrast will be noted in the analysis of gaps.

**Change made:** Introduction — added the clinician/caregiver scope-boundary sentence. (Additive; clarifies the registered scope, does not change it.)

**R2 General (c), with the two detailed nomenclature comments (on the Abstract and on the Introduction), quoted in sequence.** *Please revise the nomenclature throughout the manuscript to align with that used in the two previously published relative motion orthosis scoping reviews. Please see the nomenclature recommendations in both relative motion scoping reviews (Hirth, Howell, O’Brien 2016; Brosnan et al 2026). The preferred abbreviations are RM, RME and RMF. Please do not use RMOs. Instead write as Relative motion (RM) orthoses… I won’t continue to highlight throughout, but please change to the preferred acronyms throughout your manuscript. As per above, do not use RMO as an acronym – instead use RM orthoses or RME orthoses or RMF orthoses. Also, if adding wrist for long extensor use, use RME-plus or RMF-plus or without a wrist component use RME-only or RMF-only. This is explained in further detail in Brosnan et al, 2026. JHT in press.*

**Response.**

Adopted throughout. “RMO(s)” has been replaced with “relative motion (RM) orthosis/orthoses” (abbreviated “RM orthosis” after first use), and with “RME” or “RMF” orthoses where the injured-digit MCPJ position is relevant. In the context of postoperative long extensor or flexor tendon repair, we use RME-plus / RMF-plus where a wrist component is added and RME-only / RMF-only where it is not, following Brosnan et al. (2026). The title, abstract, keywords, main text, tables, and supporting information have been updated for consistency.

**Change made:** Whole manuscript — global nomenclature revision (RMO → RM orthosis; RME/RMF; plus/only variants). Because the change is global, with the abbreviation defined at first use, no single revised-text block is shown.

**Additional revision by the authors — orthosis description in the data extraction form (in support of R2 General (c)).** *Adopting the RME-plus/RMF-plus and RME-only/RMF-only nomenclature records whether a wrist component is present, but not how that component was configured. We have extended the extraction form to capture this descriptive detail alongside the requested classification.*

**Response.**

The plus/only nomenclature requested above is applied as the review-level classification. Because patient-perspective findings on comfort, wearability, and task compatibility may depend on how a wrist component is configured, we have extended the orthosis-details field of the data extraction form to record, where reported, the described wrist position and whether the orthosis was custom-fabricated or prefabricated. This supplements rather than replaces the requested nomenclature. The change is confined to the extraction form; no change to the manuscript text is required.

**Change made:** S3 Appendix (data extraction form), orthosis-details field — added wrist-component classification (RME-plus/RMF-plus, RME-only/RMF-only per Brosnan et al.), the described wrist position where reported, and whether the orthosis was custom-fabricated or prefabricated where reported. Because the change is confined to the extraction form, no revised-text block is shown.

**R2 detailed — Title. Reviewer #2 supplied the full title with one change (lower-case “a” → “A”):** *Full Title: Patient-perspective evidence on the acceptability, usability, and everyday impact of relative motion orthoses in adult hand rehabilitation: A scoping review protocol*

**Response.**

Adopted.

**Change made:** Title — “… a scoping review protocol” → “… A scoping review protocol” (title page, manuscript, cover letter, checklist).

**R2 detailed — structured overview of clinical applications.** *The introduction would benefit from a more structured overview of the clinical applications of RM orthoses. At present, several important diagnostic groups are omitted (e.g., sagittal band injuries, boutonnière deformity and trigger finger), while the reference list appears somewhat ad hoc. I suggest first citing the two key scoping reviews that summarise the breadth of RM orthotic applications and then cite the primary literature where each indication is listed, rather than relying on a single block of references after a broad statement.*

**Response.**

We have restructured the opening of the introduction so that the two RM scoping reviews are cited first as the syntheses mapping the breadth of RM clinical applications, followed by the principal indication groups — including sagittal band injuries, boutonnière deformity, and trigger finger — with primary literature cited against each indication.

*Replaces the sentences at lines 46–52 (“RMOs are most often discussed… clinical outcomes [1,2,4–10,12,13].”):*

**REVISED TEXT**
 The breadth of RM-orthosis clinical application has been mapped in two scoping reviews — Hirth, Howell, and O’Brien [1] and, most recently, Brosnan et al. [2]. Extensor tendon management remains the traditional indication and is supported by both general and condition-specific evidence [3–8]. Beyond it, RM orthoses are now used across a range of hand conditions, including for exercise [9], in inflammatory and degenerative conditions [10], in flexor tendon rehabilitation [3,11], for trigger finger [12–14], and in boutonnière deformity [15–17], as well as for adaptive purposes [4,10] and for sagittal band injuries, where the primary evidence is limited to earlier studies of relative-extension splinting [18–19] and no dedicated relative-motion-orthosis study has yet been reported, the available evidence being documented within the two scoping reviews [1–2].

**Change made:** Introduction — restructured opening: scoping reviews cited first, then indication groups with primary citations; added sagittal band, boutonnière, trigger finger by name.

**R2 detailed — “no comprehensive synthesis” assertion.** *This statement should acknowledge the existing work by Brosnan et al. Their scoping review includes the qualitative literature relating to patients' experiences with relative motion orthoses and is therefore directly relevant to this section. Rather than implying that no synthesis exists, I suggest discussing the Brosnan et al. review, highlighting what it has already established, and then clearly identifying the remaining gap that justifies the current review. Unless it can be demonstrated that no comprehensive synthesis exists, I would avoid this assertion. Instead, explain how the present review differs (e.g., broader inclusion criteria, updated evidence, a specific focus on patient perspectives, or additional outcomes). This would provide a clearer and more convincing rationale for the current review.*

**Response.**

Agreed — this is the core of Section A, where our submitted assertion is quoted in full. It has been removed and replaced with an acknowledgement of Brosnan et al. and a statement of the specific remaining gap: that no synthesis has yet mapped this evidence as its primary object, organised by the experiential dimensions.

*Replaces the sentences at lines 53–56 (from “By contrast, the evidence describing how adults experience…” to “…populations, and contexts.”):*

**REVISED TEXT**
 Across these applications, the literature has become increasingly informative about indications, orthosis configuration, wear schedules, and clinical outcomes. By contrast, the evidence describing how adults experience, manage, and accommodate RM orthoses in everyday life remains scattered across diverse study designs and has only recently begun to be drawn together. The updated scoping review by Brosnan et al. [2] incorporated the emerging qualitative literature and reported patient-facing findings such as comfort, appearance, adherence, and the impact of orthosis wear on daily activities. That review, however, organises evidence by clinical indication and reports patient experience within a clinically framed synthesis: because findings are grouped by the condition treated rather than by the dimension of experience described, it does not show how a single experiential dimension — such as appearance, task compatibility, wearability, daily activities, or social participation — is represented across the conditions in which RM orthoses are used, or where it remains unexamined. A synthesis arranged by condition is built to answer questions about one condition at a time — “what is known about RM orthoses for trigger finger?”, for instance — and answers them well. It is less able to answer the reverse question this review poses: taking a single aspect of experience, such as how the orthosis looks to others or how readily it lets everyday tasks be carried out, what is known about that aspect across all the conditions RM orthoses are used for, and where is it unstudied?

**Change made:** Introduction — removed the “no comprehensive synthesis” claim; replaced with the Brosnan acknowledgement and the differentiated gap statement above.

**Additional revision by the authors — abstract consistency (required by the changes above).** *Revising the introduction made two abstract sentences inaccurate: the abstract currently states that existing reviews focused on clinical outcomes “rather than patients’ everyday experiences” and that patient-perspective evidence “has not been comprehensively mapped.” Both now contradict the revised introduction, which acknowledges that Brosnan et al. reported patient experience. We have brought the abstract into line.*

**Response.**

To keep the abstract consistent with the revised introduction, we have adjusted the two abstract sentences that carried the superseded framing. The abstract cannot hold the full differentiation for reasons of length, but it no longer implies that existing reviews neglected patient experience, and no longer states that the evidence has not been comprehensively mapped; instead it states, accurately, that patient experience has not been mapped as the primary object against defined experiential domains. This mirrors the introduction and Section A. The revised abstract refers to “the most recent” review rather than citing Brosnan et al. by name, consistent with keeping citations out of the abstract.

*Replaces the two abstract sentences “Existing reviews have focused on indications, orthosis configuration, and clinical outcomes rather than patients’ everyday experiences. Relevant patient-perspective evidence is emerging but has not been comprehensively mapped.”:*

**REVISED TEXT**
Existing reviews have mapped RM-orthosis indications, configuration, and clinical outcomes, and the most recent has begun to incorporate patient-experience findings; however, patient-perspective evidence has not yet been mapped as the primary object of a synthesis, organised against defined dimensions of acceptability, usability, and everyday impact.

**Change made:** Abstract — revised the two sentences on prior reviews and mapping to match the Brosnan-aware introduction. (Required for internal consistency; no change to objective or methods.)

**R2 detailed — reference for shared decision-making / self-management. On the submitted sentence “Understanding how patients experience RMOs is also important because orthosis design decisions are increasingly being guided by principles of shared decision-making and self-management.” (line 82), Reviewer #2 wrote:** *Reference please*

**Response.**

Adopted. The sentence as submitted did not itself carry a citation: the support sat in the sentence immediately following it, which cites [4,10] (Feehan and Ewald, a five-question decision-making tool for orthosis selection; Feehan, Hoens, and Howell, partnering with people living with rheumatoid arthritis in the care of their hands). Both bear directly on shared decision-making and self-management in orthosis provision, and we have attached them to the sentence the reviewer flagged.

*Replaces the sentence at line 82, quoted in full in the comment above:*

**REVISED TEXT**
Understanding how patients experience RM orthoses is also important because orthosis design decisions are increasingly being guided by principles of shared decision-making and self-management [4,10].

**Change made:** Introduction — attached citations [4,10] to the shared-decision-making/self-management sentence, as requested. (Additive; the claim itself is unchanged.)

**R2 detailed — “clinicians lack the information needed”.** *I think this statement overstates the implications of the evidence gap. Although patient perspective evidence may not have been comprehensively mapped, clinicians do not necessarily lack the information needed to anticipate challenges, manage expectations, or deliver patient-centred orthotic care. Clinical expertise, existing qualitative evidence, and previous reviews (including Brosnan et al.) already provide valuable guidance. I suggest tempering this statement and instead emphasising that a more comprehensive synthesis would strengthen and consolidate the existing evidence base, rather than implying that clinicians are currently lacking the information needed to provide effective care.*

**Response.**

We agree and have tempered the statement.

*Replaces the sentence at lines 84–87 (“If the patient-perspective evidence base is not comprehensively mapped, clinicians lack the information needed… realities of daily life.”):*

**REVISED TEXT**
A comprehensive, patient-experience-indexed synthesis of this evidence would strengthen and consolidate the guidance already available from clinical expertise, primary qualitative research, and existing reviews, and would support more systematic, patient-centred orthotic decision-making — helping clinicians anticipate common challenges, address expectations, and tailor orthotic interventions to the realities of daily life.

**Change made:** Introduction — reworded to a strengthen-and-consolidate framing; removed the implication that clinicians currently lack the information to provide effective care.

**R2 detailed — rationale could support a comparative review; “necessary rather than optional” overstates.** *The rationale presented here could also support a broader comparative review rather than an RM orthosis-only review. If RM orthoses are hypothesised to provide a different patient experience because they are lower profile, less restrictive, and worn during active function, then comparison with other orthoses used for the same diagnoses may better demonstrate whether these differences translate into meaningful differences in acceptability, usability, and everyday impact. I suggest the authors either strengthen the justification for restricting the review to RM orthoses or acknowledge why a comparative review was not undertaken. If the authors choose to retain an RM orthosis-specific focus, I would avoid stating that such a mapping is "necessary rather than optional." While RM orthoses have features that distinguish them from other orthoses, this conclusion is stronger than the preceding argument supports. A more balanced statement would be that these unique characteristics justify or support an RM orthosis-specific synthesis. This would strengthen the rationale without overstating the need for the review.*

**Response.**

The closing-sentence changes are shown in the REVISED TEXT block under R2 General (b), above, which replaces “necessary rather than optional” with “justify and support” and adds the comparative-review-as-future-work acknowledgement. In addition, we have taken the reviewer’s point about overstatement further back into the same paragraph. On checking the underlying literature we found that the claim that RM orthoses “occupy a different experiential space” is an assumption rather than a demonstrated difference — the available head-to-head comparisons (e.g. relative motion versus metacarpophalangeal-joint-blocking orthoses for trigger finger) report broadly comparable wearability and satisfaction rather than a distinct RM experience. We have therefore reframed these sentences to rest only on what the evidence supports: that RM orthoses have distinctive design and wear characteristics whose experiential implications cannot be assumed to transfer from other orthosis types and have not yet been systematically mapped. This removes the unsupported comparative claim while preserving, and in fact strengthening, the rationale for an RM-specific synthesis.

*Replaces the two sentences beginning “They therefore occupy a different experiential space…” in the transfer-of-evidence paragraph:*

**REVISED TEXT
The experiential implications of these design and wear characteristics — for wearability, task compatibility, appearance, self-management, and participation — have not yet been systematically mapped for RM orthoses, and cannot simply be inferred from evidence generated for other orthosis types.**

**Change made:** Introduction — reframed the “different experiential space” sentences, as shown in the REVISED TEXT block immediately above. The related closing-sentence change (“necessary rather than optional” → “justify and support”) is shown in the REVISED TEXT block under R2 General (b), above.

**R2 detailed — include Brosnan in the existing-syntheses paragraph.** *Please also include the Brosnan et al. scoping review in this section and briefly explain how the current review builds on, rather than duplicates, its findings.*

**Response.**

We have done so: Brosnan et al. has been added to the “Existing evidence syntheses” paragraph as the most recent and directly relevant synthesis, with a concise build-on statement. Because this paragraph also contains three-author narrative mentions, we have taken the opportunity to apply the three-author naming convention here (see the dedicated comment below): Hirth et al. [1] and Newington et al. [11] are three-author works and are now named in full. We therefore provide the whole revised paragraph below; it also carries the revised RM-orthosis nomenclature, and its phrase “As noted above” refers back to the differentiation stated earlier in the introduction.

*Replaces the “Existing evidence syntheses” paragraph (lines 97–105, from “Existing evidence syntheses have addressed…” to “…acceptability, usability, and everyday impact.”):*

**REVISED TEXT**
Existing evidence syntheses have addressed important RM-orthosis-related questions from a different analytic standpoint. Hirth, Howell, and O’Brien [1] mapped the clinical applications and evidence base for RM orthoses across hand conditions, and Brosnan et al. [2] updated that review, incorporating the qualitative literature that has since emerged. Newington, Ross, and Howell [11] reviewed relative motion flexion orthoses with emphasis on protocols and functional outcomes. Shaw and colleagues [3] extended systematic review coverage to extensor and flexor tendon repairs, again focusing on clinical effectiveness. As noted above, the present review builds on these syntheses rather than duplicating them, and does so through three distinct analytic moves. First, it re-constitutes the evidence: where prior syntheses are organised by clinical indication, orthosis-specific patient-reported findings embedded within intervention studies — an orthosis-satisfaction rating, for example — are reported there as secondary outcomes of a clinical trial, whereas the present review extracts them and treats them as patient-perspective evidence in their own right. Second, it re-indexes that evidence by experiential dimension rather than by condition, charting it against the defined domains of acceptability, usability, and everyday impact, with no date limit and with coverage extended to grey literature. Third, this reindexing makes explicit a class of gap that a condition-organised synthesis cannot expose — where a given experiential dimension is well evidenced across conditions and where it remains unexamined — yielding a gap map that can direct future primary research and orthotic decision-making toward the dimensions and populations where evidence is currently absent. Broader upper-limb and hand-orthosis reviews have addressed satisfaction and adherence-related experiences [22–25] but were not RM-orthosis-specific and did not address the broader triad of acceptability, usability, and everyday impact.

**Change made:** Introduction — revised the whole existing-syntheses paragraph: Brosnan [2] inserted with build-on framing; Hirth [1] and Newington [11] named in full (three-author convention); nomenclature applied.

**R2 detailed — three-author citation convention.** *For citations with only three authors, the standard convention is generally to list all authors rather than using "and colleagues" (e.g., "Cole, Jamwal, and Hirth" instead of "Cole and colleagues"). Please apply this consistently throughout the manuscript. Additionally, it would improve readability if the references at the end of the sentence were grouped to align with each study type. For example, place the relevant citations immediately after "qualitative studies," "randomised trials," "case-based reports," and "survey studies" rather than listing all references together at the end. This makes it clearer which references correspond to which type of evidence.*

**Response.**

PLOS ONE uses a numbered (Vancouver) citation style, in which in-text references are numbered rather than listed by author name; narrative author naming is used only for readability. We have reviewed every narrative mention for consistency and now name all authors where a work has three authors — Hirth, Howell, and O’Brien [1] and Newington, Ross, and Howell [11] (both in the revised paragraph above), and Sekhon, Cartwright, and Francis [26] — while works with more than three authors (e.g. Brosnan et al. [2]; Shaw and colleagues [3]) retain the abbreviated form. We have also grouped the citations in the primary-literature sentence by study type; as revised, that sentence cites its sources by number alone, so no narrative naming arises within it. The two hand-therapy practice surveys [5,9] are cited in a separate sentence as evidence of how RM orthoses are prescribed and used, rather than as sources of patient-reported outcomes, so that each cited study is characterised accurately.

*Replaces the sentence at lines 106–109 (“Directly relevant primary literature includes qualitative studies by Arslan and colleagues [20] and Cole and colleagues [21]… secondary outcomes [4,6,9,12,13].”):*

**REVISED TEXT**
Directly relevant primary literature includes qualitative studies [20–21], randomised trials [7,13–14], and case-based reports [10] that report patient-reported comfort, wearability, satisfaction, or acceptability of the orthosis. Surveys of hand therapy practice further describe how RM orthoses are prescribed and used [5,9].

**Change made:** Introduction — grouped primary-literature citations by study type; applied the three-author naming convention to all narrative mentions (Hirth, Howell, and O’Brien [1]; Newington, Ross, and Howell [11]; Sekhon, Cartwright, and Francis [26]).

**R2 detailed — “heterogeneous and dispersed” and “case series”.** *This sentence makes a broad summary statement about the nature of the evidence base ("heterogeneous and dispersed") and would benefit from supporting references, unless it is explicitly intended as a synthesis of the studies cited in the preceding sentence. If it is a synthesis, consider adding the relevant citations or making the link to the preceding paragraph clearer. Additionally, "case series" is included in this list but does not appear to be supported or referenced in the preceding paragraph. Please ensure the study types are listed consistently throughout the manuscript and that the relevant references are grouped under each study type.*

**Response.**

We have made the link explicit (this sentence synthesises the studies cited immediately above) and reconciled the study-type list so that each type named is represented in the cited set. As the preceding sentence, once revised (above), cites qualitative studies, trials, case reports, and surveys, we have aligned the summary list to those types.

*Replaces the sentence at lines 109–113 (“The evidence is therefore heterogeneous and dispersed across qualitative studies, clinical trials, surveys, case reports, case series, and grey literature… suited to mapping it.”):*

**REVISED TEXT**
The evidence identified above is therefore heterogeneous and dispersed across qualitative studies, clinical trials, case-based reports, and surveys, and is likely to extend into grey literature not captured by previous reviews. The three domains of acceptability, usability, and everyday impact together provide a framework suited to mapping it.

**Change made:** Introduction — made the synthesis link explicit and reconciled the study-type list (removed unreferenced “case series”).

**R2 detailed — “For that reason” transition.** *Consider revising the opening of this paragraph. Beginning with "For that reason" is somewhat vague and does not clearly indicate which preceding point it refers to. A more direct transition (or simply starting with "A scoping review is the most appropriate approach...") would improve the flow and strengthen the rationale for the chosen review methodology.*

**Response.**

Adopted.

*Replaces the opening of the sentence at line 114 (“For that reason, a scoping review is the most appropriate approach.”):*

**REVISED TEXT**
A scoping review is the most appropriate approach for this evidence base.

**Change made:** Introduction — removed “For that reason”; paragraph now opens directly.

**R2 detailed — update the search; acknowledge Brosnan since published.** *As this is a protocol manuscript and the review has not yet been undertaken, the literature search should be updated immediately prior to study selection to ensure the evidence base is current. The preliminary search was conducted on 27 March 2026, and additional relevant publications have become available since that time. Updating the search before commencing the review is consistent with best practice and will ensure the review reflects the most current evidence. In addition, before publication of this protocol, the recently published scoping review by Brosnan et al. should be acknowledged and incorporated into the background and discussion of existing evidence syntheses. Although it was not available at the time of the preliminary search, it is now part of the published literature and is directly relevant to the rationale for the proposed review. Including it will ensure the protocol accurately reflects the current state of the evidence and clearly articulates how the proposed review will build upon the existing literature.*

**Response.**

We confirm the full database searches will be executed at the outset of operational work, immediately before formal screening begins, so the evidence base is current at the point of study selection; this is standard JBI/PRISMA-ScR practice and does not alter the registered strategy. We have added a sentence to the Search strategy stating this. Because operational work commences shortly after submission (see Study status and timeline), the search is a single event timed to screening rather than a separate later re-run. Any eligible sources published since the 27 March 2026 preliminary search — including Brosnan et al. — will be captured at this stage, screened, and reported in the completed review. Our preliminary-search paragraph is retained as an accurate point-in-time record of what was retrievable on 27 March 2026.

*To be added to the Search strategy section (after the sentence ending “…documented in the completed review), Overton, ProQuest… indexed by Embase.” at line 299):*

**REVISED TEXT**
The full database searches will be executed at the outset of operational work, immediately before formal screening begins, so that the evidence base is current at the point of study selection. Any eligible sources published since the 27 March 2026 preliminary search — including the recently published updated scoping review by Brosnan et al. [2] — will be captured at this stage, screened, and reported in the completed review.

**Change made:** Search strategy — added the sentence above, timing the full search to the outset of operational work / immediately before screening (consistent with the Study status and timeline section). Introduction — Brosnan acknowledged in the revised introduction passages shown above. Preliminary-search paragraph retained as an accurate point-in-time record. (Additive; registered strategy unchanged.)

**Additional revision by the authors — Google Scholar reproducibility.** *Although not raised by the reviewers, we identified a reproducibility limitation: the Google Scholar strategy currently reads “up to three simplified text-word combinations,” which is not reproducible as stated. We have specified the exact strings to pre-empt this.*

**Response.**

To improve reproducibility, we have specified the three Google Scholar search-string combinations in the manuscript rather than leaving them to the completed review. This does not change the search design; it makes the already-planned strategy transparent and repeatable.

*Replaces the Google Scholar parenthetical at lines 410–413 (“…Google Scholar (first 200 records sorted by relevance from up to three simplified text-word combinations, conducted in a private browsing session… documented in the completed review)…”):*

**REVISED TEXT**
Google Scholar (searched using three text-word combinations — (1) “relative motion orthosis” OR “relative motion splint”; (2) “yoke orthosis” OR “yoke splint”; (3) (“ICAM” OR “IRAM”) AND (“hand” OR “finger”) — with the first 200 records of each, sorted by relevance, screened; searches conducted in a private browsing session with the search date recorded, and the exact strings and dates reported in the completed review)

**Change made:** Search strategy — specified the three Google Scholar search strings and the first-200-records-each rule. (Additive; makes the existing strategy reproducible.)

**R2 detailed — concept exclusion criterion (generic PROs).** *I'm struggling to understand the rationale for this exclusion criterion. It seems unnecessarily restrictive to exclude generic patient-reported outcome or satisfaction measures solely because they do not explicitly reference the orthosis. Patient perspectives are often reflected through broader measures of function, satisfaction, confidence, participation, or adherence when reported in the context of an orthotic intervention. I suggest the authors provide a stronger justification for this decision and discuss the potential implications for excluding clinically relevant patient experience data. As currently framed, this criterion may also introduce selection bias by preferentially including studies that used orthosis-specific questions rather than capturing the full range of evidence relating to patients' experiences with RM orthoses.*

**Response.**

We have retained the criterion, clarified it, strengthened its justification, and added a transparency provision. The clarification: orthosis-linked patient-reported data (an orthosis-satisfaction, comfort, or wearability item referring to the orthosis) are eligible; the criterion excludes only measures that carry no orthosis-specific referent (e.g. a stand-alone DASH total or a global “satisfaction with care” score). The justification is construct validity — the review’s object is patient experience of the orthosis, and an orthosis-agnostic instrument is not, by itself, evidence about that experience; this mirrors our treatment of adherence (patient-reported adherence experiences eligible; objective wear-time metrics without patient commentary not). The transparency provision addresses the selection-bias concern.

*Replaces the sentences at lines 222–226 (“Generic patient-reported outcome or satisfaction measures (e.g. Disabilities of the Arm, Shoulder and Hand satisfaction item…) … an orthosis-specific item, comment, or theme is required.”):*

**REVISED TEXT**
Orthosis-specific patient-reported data — for example an orthosis-satisfaction rating, or a comfort or wearability item referring to the orthosis — are eligible as embedded patient-perspective evidence. Generic patient-reported outcome or satisfaction measures that carry no orthosis-specific referent (e.g. a stand-alone Disabilities of the Arm, Shoulder and Hand total, or a global “satisfaction with care” score) administered during an RM-orthosis intervention will not by themselves qualify a source for inclusion; an orthosis-specific item, comment, or theme is required. This criterion is grounded in construct validity: because the object of the review is patient experience of the orthosis, an instrument that attributes nothing to the orthosis is not, in itself, evidence about that experience. To maintain transparency, where an included source reports both orthosis-specific and generic patient-reported measures, the orthosis-specific data will be charted and the presence of the generic measures noted, so that the boundary of what was and was not captured is visible to readers. **We acknowledge that requiring an orthosis-specific referent necessarily shapes which sources enter the map, and that patient experience is sometimes conveyed through broader measures of function, satisfaction, or participation; this is a deliberate boundary of scope rather than a judgement of study quality, and recording the presence of any excluded generic measures alongside the charted orthosis-specific data keeps that boundary transparent and open to scrutiny.**

**Change made:** Concept / Types of sources — clarified eligibility of orthosis-linked data, strengthened the construct-validity justification, and added the transparency provision. Registered concept boundary unchanged.

**R2 detailed — RM orthosis definition and terminology handling.** *The definition of an RM orthosis would benefit from further clarification and supporting references. Rather than creating a new descriptive definition, I suggest adopting and referencing the terminology and definitions used in the previous RM orthosis scoping reviews. Given the inconsistent nomenclature used in the literature (e.g., relative motion, yoke, immediate relative motion, relative motion flexion/extension), it would also be helpful to explain how alternative terminology will be identified and managed during study selection. This will improve consistency with the existing literature, as well as the transparency and reproducibility of the review.*

**Response.**

We have anchored the definition to the established terminology, referencing Hirth et al. [1] and Brosnan et al. [2], and made explicit how alternative terminology is handled during selection. The Context section already lists the synonym set and states that eligibility is determined by the defining orthosis characteristic rather than by label (lines 235–245); we have added the references and a signposting sentence.

*Replaces the sentence at lines 241–245 (“Specifically, sources will be included where the orthosis has the defining feature of an RMO… left free to mobilise.”):*

**REVISED TEXT**
Consistent with the definitions used in the previous RM-orthosis scoping reviews [1–2], sources will be included where the orthosis has the defining feature of a relative motion orthosis: the metacarpophalangeal joint(s) of the affected finger or fingers are positioned in relatively greater extension or flexion than the adjacent fingers, with the interphalangeal joints left free to mobilise. Because terminology in this literature is inconsistent, eligibility is determined by this defining characteristic rather than by the label used; sources using any of the terms listed above, or related variants, are assessed against this characteristic at screening, and the search strategy incorporates the full range of these synonyms, eponyms, and device-name variants (S2 Appendix).

**Change made:** Context — anchored the definition to Hirth [1] and Brosnan [2] and added the terminology-handling signpost. (S2 Appendix already contains the synonym set.)

**R2 detailed — “accessible English translation”.** *Please clarify what is meant by "accessible English translation," as this term is ambiguous and may be interpreted differently by readers.*

**Response.**

We have defined the term at its first occurrence in the Types of sources section. The phrase appears once more, in the Search strategy section; as the term is now defined at first use, that later mention is unchanged.

*Replaces the sentence at lines 264–266 (“Only sources available in English, or with an accessible English translation, will be included… to the review team.”):*

**REVISED TEXT**
Only sources available in English, or for which an accessible English translation can be obtained, will be included in the final review. By “accessible English translation” we mean a full-text English version that is either already published, or that can be produced reliably using the resources available to the review team; sources available only as an English-language abstract, without a full text that can be read or translated in full, will be recorded and reported as excluded for language. This language limit reflects the review team’s resource and translation capacity and will be considered when interpreting the geographical and contextual distribution of the evidence.

**Change made:** Types of sources — defined “accessible English translation” and specified how language-excluded sources are recorded and reported.

**D. Summary**

We have adopted all three of Reviewer #1’s suggestions and all of Reviewer #2’s comments, either by direct change or, where we have retained a design decision (the RM-orthosis-specific scope and the concept exclusion criterion), by strengthening the justification and adding transparency provisions as the reviewers invited. Engaging with Brosnan et al. (2026) has materially improved the manuscript by sharpening how the proposed review complements and builds on the existing RM syntheses. We thank the editor and reviewers again for their constructive input and are happy to make further revisions if required.
