# Supplementary material for: Patient-perspective evidence on the acceptability, usability, and everyday impact of relative motion orthoses in adult hand rehabilitation: A scoping review protocol
Source: PLoS One. 2026 Sep 11;21(9):e0349852. doi: 10.1371/journal.pone.0349852 (PMC13567770; doi:10.1371/journal.pone.0349852)
Supplement: Supplementary file 6 [file pone.0349852.s004.docx]

In-confidence review --- Manuscript Number: PONE-D-26-20165

Article Type: Study Protocol

Full Title: Patient-perspective evidence on the acceptability, usability, and everyday impact of relative motion orthoses in adult hand rehabilitation: a scoping review protocol

General comments:

The recent scoping review on relative motion orthoses should be incorporated into this study protocol. The authors should clearly acknowledge this review and explain how the proposed review builds on, rather than duplicates, the existing evidence base.

In my opinion, the proposed review question is overly narrow and may not address the questions that are most relevant to clinicians. Relative motion orthoses are typically one of several orthotic options used for the same diagnosis or therapeutic purpose, yet comparator orthoses are excluded without sufficient justification. For example, trigger finger may involve MCPJ-blocking orthoses; extensor tendon rehabilitation may involve wrist-hand-finger orthoses (WHFOs) with the IP joints free, relative motion approaches, or Norwich Regime; and flexor tendon rehabilitation may involve dorsal blocking, Manchester Short, or other protocols etc. A broader comparative synthesis would likely provide greater clinical value by allowing therapists to compare the acceptability, usability, and everyday impact of the orthotic options commonly considered for the same clinical presentation. If the authors elect to retain the current review scope, the rationale for excluding comparator orthoses should be strengthened.

Please revise the nomenclature throughout the manuscript to align with that used in the two previously published relative motion orthosis scoping reviews.

| Existing/Submitted | Feedback/Recommendation |
| --- | --- |
| Full Title: Patient-perspective evidence on the acceptability, usability, and everyday impact of relative motion orthoses in adult hand rehabilitation: a scoping review protocol | Full Title: Patient-perspective evidence on the acceptability, usability, and everyday impact of relative motion orthoses in adult hand rehabilitation: A scoping review protocol |
| Abstract: Relative motion orthoses (RMOs) are finger-based orthoses used in adult hand rehabilitation to protect healing structures or optimise movement patterns while permitting active hand use. Existing reviews have focused on indications, orthosis configuration, and clinical outcomes rather than patients' everyday experiences. Relevant patient-perspective evidence is emerging but has not been comprehensively mapped. The objective of this scoping review is to map the extent, range, and nature of patient perspective evidence on the acceptability, usability, and everyday impact of RMOs in adult hand rehabilitation. The review will follow Joanna Briggs Institute methodology for scoping reviews and be reported using the Preferred Reporting Items for Systematic Reviews and Meta Analyses extension for Scoping Reviews. Sources will include adults aged 18 years or older who have used an RMO for hand rehabilitation. Any adult hand rehabilitation context will be considered. Qualitative, quantitative, mixed-methods, case-based, survey, and grey literature sources will be included when patient-perspective data can be extracted. Ovid MEDLINE, Embase, CINAHL Complete, PsycINFO, Scopus, and Web of Science Core Collection will be searched, together with Google Scholar, ProQuest Dissertations and Theses Global, and Overton. Two reviewers will independently screen and extract data, with a third reviewer available for adjudication. Critical appraisal will not be undertaken. The protocol has been prospectively registered on the Open Science Framework (https://osf.io/dcyf8/overview). Findings will be presented in tabular, diagrammatic, and narrative form, including a summary-of characteristics table and a concept-mapping matrix. This review will provide an evidence map to guide patient-centred orthotic practice and identify research priorities. | Please see the nomenclature recommendations in both relative motion scoping reviews (Hirth, Howell, O’Brien 2016; Brosnan et al 2026). The preferred abbreviations are RM, RME and RMF. Please do not use RMOs. Instead write as Relative motion (RM) orthoses…  I won’t continue to highlight throughout, but please change to the preferred acronyms throughout your manuscript. |
| Introduction –  Relative motion orthoses (RMOs) are an established component of contemporary hand rehabilitation | As per above, do not use RMO as an acronym – instead use RM orthoses or RME orthoses or RMF orthoses. Also, if adding wrist for long extensor use, use RME-*plus* or RMF-*plus* or without a wrist component use RME-*only* or RMF-*only*. This is explained in further detail in Brosnan et al, 2026. JHT in press. |
| RMOs are most often discussed in relation to extensor tendon management, but their use now extends beyond that traditional indication to flexor tendon rehabilitation, exercise applications, inflammatory and degenerative conditions, and other hand-therapy problems [1–13]. | The introduction would benefit from a more structured overview of the clinical applications of RM orthoses. At present, several important diagnostic groups are omitted (e.g., sagittal band injuries, boutonnière deformity and trigger finger), while the reference list appears somewhat ad hoc. I suggest first citing the two key scoping reviews that summarise the breadth of RM orthotic applications and then cite the primary literature where each indication is listed, rather than relying on a single block of references after a broad statement. |
| By contrast, the evidence describing how adults experience, manage, and accommodate RMOs in everyday life remains scattered, heterogeneous, and incompletely mapped. Despite this emerging literature, there has been no comprehensive synthesis mapping the extent, range, and nature of patient-perspective evidence on RMO use across study designs, populations, and contexts. | This statement should acknowledge the existing work by Brosnan et al. Their scoping review includes the qualitative literature relating to patients' experiences with relative motion orthoses and is therefore directly relevant to this section. Rather than implying that no synthesis exists, I suggest discussing the Brosnan et al. review, highlighting what it has already established, and then clearly identifying the remaining gap that justifies the current review. Unless it can be demonstrated that no comprehensive synthesis exists, I would avoid this assertion. Instead, explain how the present review differs (e.g., broader inclusion criteria, updated evidence, a specific focus on patient perspectives, or additional outcomes). This would provide a clearer and more convincing rationale for the current review. |
| Understanding how patients experience RMOs is also important because orthosis design decisions are increasingly being guided by principles of shared decision-making and self-management. | Reference please |
| If the patient perspective evidence base is not comprehensively mapped, clinicians lack the information needed to anticipate common challenges, address expectations, and tailor orthotic interventions to the realities of daily life. | I think this statement overstates the implications of the evidence gap. Although patient perspective evidence may not have been comprehensively mapped, clinicians do not necessarily lack the information needed to anticipate challenges, manage expectations, or deliver patient-centred orthotic care. Clinical expertise, existing qualitative evidence, and previous reviews (including Brosnan et al.) already provide valuable guidance. I suggest tempering this statement and instead emphasising that a more comprehensive synthesis would strengthen and consolidate the existing evidence base, rather than implying that clinicians are currently lacking the information needed to provide effective care. |
| However, it cannot be assumed that findings from broader orthosis literature transfer directly to RMOs. RMOs have a lower profile than many dynamic or forearm-based static orthoses, are frequently used during active hand function rather than at rest, and may be worn across a range of real-world contexts and diagnostic groups [1–13]. They therefore occupy a different experiential space from bulkier, more restrictive, or more overtly immobilising devices. Questions of wearability, task compatibility, appearance, self-management, and participation may consequently take a different form in RMO use than in other orthotic contexts. This makes RMO-specific mapping of patient perspective evidence necessary rather than optional. | The rationale presented here could also support a broader comparative review rather than an RM orthosis-only review. If RM orthoses are hypothesised to provide a different patient experience because they are lower profile, less restrictive, and worn during active function, then comparison with other orthoses used for the same diagnoses may better demonstrate whether these differences translate into meaningful differences in acceptability, usability, and everyday impact. I suggest the authors either strengthen the justification for restricting the review to RM orthoses or acknowledge why a comparative review was not undertaken.  If the authors choose to retain an RM orthosis-specific focus, I would avoid stating that such a mapping is "necessary rather than optional." While RM orthoses have features that distinguish them from other orthoses, this conclusion is stronger than the preceding argument supports. A more balanced statement would be that these unique characteristics justify or support an RM orthosis-specific synthesis. This would strengthen the rationale without overstating the need for the review. |
| Existing evidence syntheses have addressed important RMO-related questions from a different analytic standpoint. Hirth and colleagues [1] mapped the clinical applications and evidence base for RMOs across hand conditions. Newington and colleagues [10] reviewed relative motion flexion orthoses with emphasis on protocols and functional outcomes. Shaw and colleagues [2] extended systematic review coverage to extensor and flexor tendon repairs, again focusing on clinical effectiveness. Broader upper-limb and hand-orthosis reviews have addressed satisfaction and adherence-related experiences [14–17] but were not RMO-specific and did not address the broader triad of acceptability, usability, and everyday impact. | Please also include the Brosnan et al. scoping review in this section and briefly explain how the current review builds on, rather than duplicates, its findings. |
| Directly relevant primary literature includes qualitative studies by Arslan and colleagues [20] and Cole and colleagues [21], alongside randomised trials, case-based reports, and survey studies that report patient-reported comfort, wearability, satisfaction, or acceptability of the orthosis as secondary outcomes [4,6,9,12,13]. | For citations with only three authors, the standard convention is generally to list all authors rather than using "and colleagues" (e.g., "Cole, Jamwal, and Hirth" instead of "Cole and colleagues"). Please apply this consistently throughout the manuscript. Additionally, it would improve readability if the references at the end of the sentence were grouped to align with each study type. For example, place the relevant citations immediately after "qualitative studies," "randomised trials," "case-based reports," and "survey studies" rather than listing all references together at the end. This makes it clearer which references correspond to which type of evidence. |
| The evidence is therefore heterogeneous and dispersed across qualitative studies, clinical trials, surveys, case reports, case series, and grey literature, and the three domains of acceptability, usability, and everyday impact together provide a framework suited to mapping it. | This sentence makes a broad summary statement about the nature of the evidence base ("heterogeneous and dispersed") and would benefit from supporting references, unless it is explicitly intended as a synthesis of the studies cited in the preceding sentence. If it is a synthesis, consider adding the relevant citations or making the link to the preceding paragraph clearer.  Additionally, "case series" is included in this list but does not appear to be supported or referenced in the preceding paragraph. Please ensure the study types are listed consistently throughout the manuscript and that the relevant references are grouped under each study type. |
| For that reason, a scoping review is the most appropriate approach. Scoping reviews are particularly suited to bodies of evidence that are emerging, conceptually broad, methodologically diverse, and not yet sufficiently mature or standardised for a focused effectiveness review [22–25]. In the present topic area, the purpose is not to estimate pooled effects or judge intervention efficacy, but to determine what patient-perspective evidence exists, how that evidence has been generated, what dimensions of acceptability, usability, and everyday impact have been addressed, and where important gaps remain. | Consider revising the opening of this paragraph. Beginning with "For that reason" is somewhat vague and does not clearly indicate which preceding point it refers to. A more direct transition (or simply starting with "A scoping review is the most appropriate approach...") would improve the flow and strengthen the rationale for the chosen review methodology. |
| A preliminary search of Ovid MEDLINE, the Cochrane Database of Systematic Reviews, PROSPERO, the Joanna Briggs Institute (JBI) Evidence Synthesis journal, and the Open Science Framework (OSF) was conducted on 27 March 2026. Relevant syntheses were identified [1,2,10,14–17]. However, no scoping review, systematic review, or registered protocol mapping patient-perspective evidence on the acceptability, usability, and everyday impact of RMOs in adult hand rehabilitation was retrieved. | As this is a protocol manuscript and the review has not yet been undertaken, the literature search should be updated immediately prior to study selection to ensure the evidence base is current. The preliminary search was conducted on 27 March 2026, and additional relevant publications have become available since that time. Updating the search before commencing the review is consistent with best practice and will ensure the review reflects the most current evidence.  In addition, before publication of this protocol, the recently published scoping review by Brosnan et al. should be acknowledged and incorporated into the background and discussion of existing evidence syntheses. Although it was not available at the time of the preliminary search, it is now part of the published literature and is directly relevant to the rationale for the proposed review. Including it will ensure the protocol accurately reflects the current state of the evidence and clearly articulates how the proposed review will build upon the existing literature. |
| Concept –  General functional outcomes without an explicit orthosis-experience component will not be treated as patient-perspective evidence. Generic patient-reported outcome or satisfaction measures (e.g. Disabilities of the Arm, Shoulder and Hand satisfaction item, global satisfaction with care) administered during an RMO intervention without an item specifically referencing the orthosis will not by themselves qualify a source for inclusion; an orthosis-specific item, comment, or theme is required. | I'm struggling to understand the rationale for this exclusion criterion. It seems unnecessarily restrictive to exclude generic patient-reported outcome or satisfaction measures solely because they do not explicitly reference the orthosis. Patient perspectives are often reflected through broader measures of function, satisfaction, confidence, participation, or adherence when reported in the context of an orthotic intervention. I suggest the authors provide a stronger justification for this decision and discuss the potential implications for excluding clinically relevant patient experience data. As currently framed, this criterion may also introduce selection bias by preferentially including studies that used orthosis-specific questions rather than capturing the full range of evidence relating to patients' experiences with RM orthoses. |
| Context –  Specifically, sources will be included where the orthosis has the defining feature of an RMO: the metacarpophalangeal joints of the affected finger or fingers are positioned in relatively greater extension or flexion than the adjacent fingers, with the interphalangeal joints left free to mobilise. No geographical limits will be applied. | The definition of an RM orthosis would benefit from further clarification and supporting references. Rather than creating a new descriptive definition, I suggest adopting and referencing the terminology and definitions used in the previous RM orthosis scoping reviews. Given the inconsistent nomenclature used in the literature (e.g., relative motion, yoke, immediate relative motion, relative motion flexion/extension), it would also be helpful to explain how alternative terminology will be identified and managed during study selection. This will improve consistency with the existing literature, as well as the transparency and reproducibility of the review. |
| Types of sources –  Only sources available in English, or with an accessible English translation, will be included in the final review, reflecting the resource and translation capacity available to the review team. | Please clarify what is meant by "accessible English translation," as this term is ambiguous and may be interpreted differently by readers. |
