# Supplementary material for: Patient-perspective evidence on the acceptability, usability, and everyday impact of relative motion orthoses in adult hand rehabilitation: A scoping review protocol
Source: PLoS One. 2026 Sep 11;21(9):e0349852. doi: 10.1371/journal.pone.0349852 (PMC13567770; doi:10.1371/journal.pone.0349852)
Supplement: Supplementary file 7 [file pone.0349852.s006.docx]

**Response to Reviewers — Revision 2**

**PLOS ONE | Manuscript PONE-D-26-20165**

Patient-perspective evidence on the acceptability, usability, and everyday impact of relative motion orthoses in adult hand rehabilitation: A scoping review protocol

We thank the Academic Editor and both reviewers for their assessment of the revised protocol. We are grateful to Reviewer #1 for recommending acceptance, and to Reviewer #2 for supporting the protocol for publication. Reviewer #2 made one final formatting request, which we have implemented in full. No other changes have been made.

**Reviewer #2**

**Comment**

*“A final request is to please update lines 51 and 52 ‘…repairs, the terms RME-plus and RMF-plus denote the addition of a wrist component and RME-only and RMF-only its absence, following Brosnan et al. [2].’ to have the plus and only in italics as per Brosnan et al. Please also put the only and plus in italics throughout your manuscript.”*

**Response**

Done. We have set the suffixes *plus* and *only* in italics within the compound terms RME-*plus*, RMF-*plus*, RME-*only*, and RMF-*only*, matching the styling used by Brosnan et al. These terms appear in the manuscript only in the defining sentence at lines 51–52, where the styling has been applied; it has also been applied to the same terms in the S3 data-extraction appendix, so that the convention is consistent across the submission. No wording was changed; this is a typographical correction only.

**Reviewer #1**

We thank Reviewer #1 for recommending acceptance and for confirming that the points raised at the first round were satisfactorily addressed. No further changes were requested, and none beyond the formatting correction above have been made.
