# Supplementary material for: Patient-perspective evidence on the acceptability, usability, and everyday impact of relative motion orthoses in adult hand rehabilitation: A scoping review protocol
Source: PLoS One. 2026 Sep 11;21(9):e0349852. doi: 10.1371/journal.pone.0349852 (PMC13567770; doi:10.1371/journal.pone.0349852)
Supplement: Peer review history

# Peer review: Decision Letter 0

**DOI:** 10.1371/journal.pone.0349852.r001

**Authors:** Emma Campbell

8 Jul 2026

Dear Dr. Chockalingam,

Thank you for submitting your manuscript to PLOS ONE. After careful consideration, we feel that it has merit but does not fully meet PLOS ONE’s publication criteria as it currently stands. Therefore, we invite you to submit a revised version of the manuscript that addresses the points raised during the review process.

Please submit your revised manuscript by Aug 22 2026 11:59PM. If you will need more time than this to complete your revisions, please reply to this message or contact the journal office at plosone@plos.org. When you're ready to submit your revision, log on to https://www.editorialmanager.com/pone/ and select the 'Submissions Needing Revision' folder to locate your manuscript file.

- A letter that responds to each point raised by the academic editor and reviewer(s). You should upload this letter as a separate file labeled 'Response to Reviewers'.
- A marked-up copy of your manuscript that highlights changes made to the original version. You should upload this as a separate file labeled 'Revised Manuscript with Track Changes'.
- An unmarked version of your revised paper without tracked changes. You should upload this as a separate file labeled 'Manuscript'.

If you would like to make changes to your financial disclosure, please include your updated statement in your cover letter. Guidelines for resubmitting your figure files are available below the reviewer comments at the end of this letter.

If applicable, we recommend that you deposit your laboratory protocols in protocols.io to enhance the reproducibility of your results. Protocols.io assigns your protocol its own identifier (DOI) so that it can be cited independently in the future. For instructions see: https://journals.plos.org/plosone/s/submission-guidelines#loc-laboratory-protocols. Additionally, PLOS ONE offers an option for publishing peer-reviewed Lab Protocol articles, which describe protocols hosted on protocols.io. Read more information on sharing protocols at https://plos.org/protocols?utm_medium=editorial-email&utm_source=authorletters&utm_campaign=protocols.

As the corresponding author, your ORCID iD is verified in the submission system and will appear in the published article. PLOS supports the use of ORCID, and we encourage all coauthors to register for an ORCID iD and use it as well. Please encourage your coauthors to verify their ORCID iD within the submission system before final acceptance, as unverified ORCID iDs will not appear in the published article. *Only*  the individual author can complete the verification step; PLOS staff *cannot*  verify ORCID iDs on behalf of authors.

We look forward to receiving your revised manuscript.

Kind regards,

Emma Campbell, Ph.D

Staff Editor

PLOS One

Journal Requirements:

When submitting your revision, we need you to address these additional requirements.

1.Please ensure that your manuscript meets PLOS ONE's style requirements, including those for file naming. The PLOS ONE style templates can be found at

https://journals.plos.org/plosone/s/file?id=wjVg/PLOSOne_formatting_sample_main_body.pdf and

https://journals.plos.org/plosone/s/file?id=ba62/PLOSOne_formatting_sample_title_authors_affiliations.pdf

2. If the reviewer comments include a recommendation to cite specific previously published works, please review and evaluate these publications to determine whether they are relevant and should be cited. There is no requirement to cite these works unless the editor has indicated otherwise.

[Note: HTML markup is below. Please do not edit.]

Reviewers' comments:

Reviewer's Responses to Questions

**Comments to the Author**

1. Does the manuscript provide a valid rationale for the proposed study, with clearly identified and justified research questions?

Reviewer #1: Yes

Reviewer #2: Partly

**********

2. Is the protocol technically sound and planned in a manner that will lead to a meaningful outcome and allow testing the stated hypotheses?

Reviewer #1: Yes

Reviewer #2: Partly

**********

3. Is the methodology feasible and described in sufficient detail to allow the work to be replicable?

Reviewer #1: Yes

Reviewer #2: Yes

**********

4. Have the authors described where all data underlying the findings will be made available when the study is complete?

The PLOS Data policy

Reviewer #1: Yes

Reviewer #2: Yes

**********

5. Is the manuscript presented in an intelligible fashion and written in standard English?

Reviewer #1: Yes

Reviewer #2: Yes

**********

Please use the space provided to explain your answers to the questions above and, if applicable, provide comments about issues authors must address before this protocol can be accepted for publication. You may also include additional comments for the author, including concerns about research or publication ethics.

You may also provide optional suggestions and comments to authors that they might find helpful in planning their study.

Reviewer #1: Reviewer Comments

This is a well-written and methodologically rigorous scoping review protocol addressing an important and underexplored aspect of relative motion orthosis (RMO) use. The rationale is well developed, the review questions are clearly articulated, and the protocol demonstrates strong adherence to JBI and PRISMA-ScR guidance. The eligibility criteria, search strategy, and data extraction procedures are described in considerable detail, which should enhance transparency and reproducibility. The topic is clinically relevant and addresses an important gap in the current literature. I have only a few minor suggestions that may further strengthen the protocol.

Comment 1. Clarification of timing of patient-perspective outcomes

The protocol appropriately identifies the timing of patient-perspective data collection as a data extraction item. However, patient experiences such as comfort, satisfaction, usability, adherence-related experiences, and everyday impact may differ substantially according to the stage of orthosis use (e.g., immediately after fitting, during the adaptation period, or after long-term use).

It would be helpful if the authors could clarify how timing-related information will be synthesized and presented in the final review. For example, will findings be grouped according to stages of orthosis use where sufficient data are available? Such clarification may enhance the interpretability of the resulting evidence map.

Comment 2. Further justification for an RMO-specific review

The rationale for an RMO-specific review is generally convincing. However, given that several recent qualitative and patient-experience studies have emerged in this area, the authors may wish to further highlight how the growing patient-perspective evidence base has created the need for a dedicated synthesis. This may help further distinguish the current review from previous RMO reviews that primarily focused on clinical outcomes and orthosis application.

Comment 3. Transparency of coding decisions across conceptual domains

The conceptual framework of acceptability, usability, and everyday impact is a notable strength of the protocol. However, several patient-reported constructs (e.g., comfort, satisfaction, wearability, and adherence-related experiences) may reasonably overlap across domains.

Although the protocol indicates that multi-domain coding will be used when appropriate, providing additional coding examples or decision rules within the extraction framework may further improve transparency and reproducibility. This may be particularly useful when different reviewers are required to classify findings that could plausibly fit more than one conceptual category.

Overall Recommendation

Minor Revision

The protocol is methodologically sound, addresses an important evidence gap, and is likely to make a valuable contribution to the hand rehabilitation literature. The suggested revisions are intended to improve clarity and interpretability rather than to address major methodological concerns.

Reviewer #2: Please also see attachment -

General comments:

The recent scoping review on relative motion orthoses should be incorporated into this study protocol. The authors should clearly acknowledge this review and explain how the proposed review builds on, rather than duplicates, the existing evidence base.

In my opinion, the proposed review question is overly narrow and may not address the questions that are most relevant to clinicians. Relative motion orthoses are typically one of several orthotic options used for the same diagnosis or therapeutic purpose, yet comparator orthoses are excluded without sufficient justification. For example, trigger finger may involve MCPJ-blocking orthoses; extensor tendon rehabilitation may involve wrist-hand-finger orthoses (WHFOs) with the IP joints free, relative motion approaches, or Norwich Regime; and flexor tendon rehabilitation may involve dorsal blocking, Manchester Short, or other protocols etc. A broader comparative synthesis would likely provide greater clinical value by allowing therapists to compare the acceptability, usability, and everyday impact of the orthotic options commonly considered for the same clinical presentation. If the authors elect to retain the current review scope, the rationale for excluding comparator orthoses should be strengthened.

Please revise the nomenclature throughout the manuscript to align with that used in the two previously published relative motion orthosis scoping reviews.

**********

what does this mean?). If published, this will include your full peer review and any attached files.

If you choose “no”, your identity will remain anonymous but your review may still be made public.

**Do you want your identity to be public for this peer review?**  For information about this choice, including consent withdrawal, please see our Privacy Policy

Reviewer #1: No

Reviewer #2: No

**********

[NOTE: If reviewer comments were submitted as an attachment file, they will be attached to this email and accessible via the submission site. Please log into your account, locate the manuscript record, and check for the action link "View Attachments". If this link does not appear, there are no attachment files.]

To ensure your figures meet our technical requirements, please review our figure guidelines: https://journals.plos.org/plosone/s/figures

You may also use PLOS’s free figure tool, NAAS, to help you prepare publication quality figures: https://journals.plos.org/plosone/s/figures#loc-tools-for-figure-preparation.

NAAS will assess whether your figures meet our technical requirements by comparing each figure against our figure specifications.

Attachment Submitted filename: PLOS_July 2026.docx

# Author comment: Author response to Decision Letter 1

**DOI:** 10.1371/journal.pone.0349852.r002

23 Jul 2026

Response to reviewers file provides a detailed reply. The same is being attached here as well.

Response to Reviewers

PLOS ONE | Manuscript PONE-D-26-20165

Patient-perspective evidence on the acceptability, usability, and everyday impact of relative motion orthoses in adult hand rehabilitation: A scoping review protocol

We thank the Staff Editor and both reviewers for their careful and constructive assessment of our protocol. We are grateful for Reviewer #1’s positive appraisal and minor-revision recommendation, and for Reviewer #2’s detailed comments, which have materially strengthened the manuscript. The most significant development since submission is the publication of the updated scoping review by Brosnan et al. (2026), which appeared after our preliminary search; we have engaged with it fully. Because several comments from both reviewers relate to it, we set out our position once, in Section A, and refer back to it where relevant.

For each point, we give our response and, where the change is a specific wording change, the exact revised text in a shaded “REVISED TEXT” block that can be read directly against the manuscript. Reviewer and editor comments are reproduced verbatim in italics; the short headings and the R1/R2 numbering are ours, added for navigation only. Where a comment is brief, we identify the submitted sentence it was attached to. Revisions we have made on our own initiative are headed “Additional revision by the authors.” Line references are to the submitted manuscript.

Note on references: Brosnan et al. (2026) was published after submission and therefore does not appear in the submitted manuscript. In the revised manuscript it is added as a new reference, integrated into the reference list in order of first appearance and cited as [2], immediately after Hirth et al. [1]; all references that previously followed Hirth et al. are shifted down by one position accordingly, and the reference list is renumbered in order of first appearance. The full citation is: Brosnan M, Hirth MJ, O’Riordan C, Morrissey A-M. Ten years on: an updated scoping review of relative motion orthoses for managing various hand conditions. J Hand Ther. 2026 (in press). doi:10.1016/j.jht.2026.02.006.

A. How the proposed review relates to Brosnan et al. (2026) — our central position

This underlies Reviewer #1’s comment R1.2 and several of Reviewer #2’s comments; we state it here and cross-reference it throughout.

Brosnan et al. (2026), an update of Hirth et al. (2016), appeared after our preliminary search of 27 March 2026 and was not available at drafting. We agree it is directly relevant and have revised the manuscript to acknowledge and engage with it. We first correct our own framing. Our submitted text asserted that “there has been no comprehensive synthesis mapping the extent, range, and nature of patient-perspective evidence on RMO use across study designs, populations, and contexts” (lines 53–56). With Brosnan et al. now published, that assertion is no longer defensible as written, and we have removed it. In its place the revised introduction makes the claim we can substantiate: that no synthesis has yet mapped patient-perspective evidence on RM orthosis use as its primary object, organised by the experiential dimensions of acceptability, usability, and everyday impact. This is a more precise claim than the one we submitted, not a lesser one: it names the gap this review is designed to close, it is verifiable against both published reviews (the four axes below), and the manuscript sets out why closing it matters — an orthosis delivers its benefit only if the patient wears and manages it in daily life, so acceptability, usability, and everyday impact are preconditions of its effectiveness rather than incidental to it. Brosnan et al. appraise four studies containing qualitative data (the interpretive qualitative studies of Arslan et al. and Cole et al.; the qualitative case report of Feehan et al.; and the mixed-methods study of Mottay et al.) and report patient-facing findings including comfort, skin effects, appearance, adherence, expectations, and the impact of orthosis wear on daily activities and emotional and social life. We do not claim these experiences are undocumented, but reporting them within an indication-organised synthesis is not the same as charting them as evidence in their own right.

Our review is distinct and complementary, along four axes verifiable against the two published reviews and our own protocol:

1. Object and organisation. Brosnan et al. and Hirth et al. organise the evidence by clinical indication (which conditions RME/RMF orthoses are used for; level of evidence per condition; recommended MCPJ differential; preferred design). Brosnan et al. present their qualitative and patient-experience material within these indication categories (protective, exercise, adaptive) and draw it together narratively in their discussion. Our review makes patient-perspective evidence the sole object of the map and charts it against three pre-specified, operationally defined domains — acceptability (informed by Sekhon et al.), usability, and everyday impact (informed by the ICF) — with a concept-mapping matrix as its primary output. The difference is the mode of organisation: because Brosnan et al. index by indication and synthesise experience narratively, their account does not systematically chart how a single dimension of experience — appearance, task compatibility, wearability, sleep, or social participation — is represented across conditions, or where such evidence is absent. A map indexed by experiential domain is built to make those cross-diagnosis patterns, and their gaps, explicit and systematic rather than narrative.

2. What is extracted from shared sources. Brosnan et al. include primary intervention studies and report the patient-reported outcomes within them (for example orthosis satisfaction, reported alongside range of motion and pain) as clinical outcomes, organised by indication. Our review takes the same class of source but extracts and charts those orthosis-specific patient-reported findings — orthosis-satisfaction ratings, comfort, wearability, and adherence-related experience — as patient-perspective evidence in their own right, against the acceptability/usability/everyday-impact framework. The difference is not which sources are eligible but what is extracted from them and how it is charted: data that appear in Brosnan et al. as secondary clinical endpoints are, in our review, treated as primary units of a patient-experience map. (For accuracy: Brosnan et al. did exclude secondary source types — cross-sectional studies, narrative and systematic reviews, and clinical guidelines — per their Table 2; our point on this axis concerns the primary studies common to both reviews.)

3. Source breadth and time coverage. Brosnan et al. searched four databases (MEDLINE, Embase, PubMed, CINAHL), excluded grey literature, and applied a November 2015–August 2025 window. Hirth et al. (2016) covered the earlier literature, but the patient-facing content it captured appears there as effectiveness outcomes, orthosis-design observations, or expert opinion — not as patient-perspective evidence charted against an experiential framework — and that earlier literature was not revisited by Brosnan et al. (whose criteria excluded any publication included in the 2016 review). Our review searches six databases (Ovid MEDLINE, Embase, CINAHL Complete, PsycINFO, Scopus, Web of Science Core Collection), and the additions of PsycINFO, Scopus, and Web of Science specifically index the patient-experience and psychosocial literature central to this review; it also applies no date limit and admits grey literature and theses. Its included set and charted content are therefore expected to extend beyond, rather than replicate, Brosnan et al.

4. Analytic output. Our concept-mapping matrix codes each source as primary, embedded/secondary, or incidental against the three domains, producing an explicit map of where patient-experience evidence is concentrated and where it is absent across populations, diagnoses, methods, and conceptual domains. This gap map has a downstream value neither prior review provides: by making the shape of the evidence — and its holes — explicit, it allows future primary research to be targeted at genuinely under-evidenced domains and populations rather than duplicating well-covered ground, and it signals to clinicians where current practice necessarily rests on extrapolation beyond the available patient-perspective evidence. Neither prior review produced an equivalent artefact.

We are candid that the small existing body of dedicated qualitative RM-orthosis research — the studies by Arslan, Cole, Feehan, and Mottay — is already included in Brosnan et al., and our contribution therefore lies in the analytic lens and the breadth of additional source types rather than in identifying previously undiscovered studies. We are equally candid on the clinical rationale: the prior reviews and the primary qualitative studies already provide clinicians with substantive experiential guidance, and we do not claim to supply knowledge those sources lack. What the present review adds is structure — a patient-experience-indexed map with an explicit gap analysis — that allows researchers to locate the experiential evidence relevant to a given dimension of use and to target future study at under-evidenced domains, and allows clinicians to recognise where that evidence is thin or absent and current practice therefore rests on extrapolation. This directly addresses the reviewers’ shared request to show how the review builds on, rather than duplicates, the existing syntheses.

We note PLOS ONE’s guidance that there is no obligation to cite reviewer-recommended works; we cite and engage Brosnan et al. because it is genuinely central to the rationale.

B. Response to Reviewer #1

We thank Reviewer #1 for the positive appraisal and for three suggestions, all adopted.

R1.1 (timing of patient-perspective outcomes). The protocol appropriately identifies the timing of patient-perspective data collection as a data extraction item. However, patient experiences such as comfort, satisfaction, usability, adherence-related experiences, and everyday impact may differ substantially according to the stage of orthosis use (e.g., immediately after fitting, during the adaptation period, or after long-term use). It would be helpful if the authors could clarify how timing-related information will be synthesized and presented in the final review. For example, will findings be grouped according to stages of orthosis use where sufficient data are available? Such clarification may enhance the interpretability of the resulting evidence map.

Response.

We agree. The protocol already records timing of patient-perspective data collection as a charting item (Data extraction, lines 322–323). We have added to the Data analysis and presentation section a sentence specifying that, where data allow, findings will be grouped and presented by stage of orthosis use, and that where sources do not permit staging this will be recorded as a characteristic of the evidence and noted as a gap. Where sources report elapsed time (weeks since injury or surgery, or since the orthosis was first applied), this will be used to assign stage, so that grouping rests on reported timing rather than on stage labels alone; the data extraction form (S3 Appendix) has been updated accordingly to record elapsed time where available.

To be added to the Data analysis and presentation section (after the sentence ending “…(multi-domain coding).” at line 352):

REVISED TEXT

Where sufficient data are available, patient-perspective findings will also be grouped and presented by stage of orthosis use — for example early or fitting stage, adaptation stage, and longer-term or established wear — to reflect that acceptability, usability, and everyday impact may differ across the course of orthosis use. Where sources report elapsed time in weeks since injury, surgery, or first application, this will be used to assign stage, so that grouping rests on reported timing rather than labels alone. Where sources do not report the timing of patient-perspective data, this will be recorded as a characteristic of the evidence and noted as a gap.

Change made: Data analysis and presentation — added the stage-of-use grouping sentence above. S3 Appendix (data extraction form), Timing field — added capture of elapsed time where reported (weeks since injury or surgery, and weeks since the orthosis was first applied), and the rule that stage of orthosis use is assigned from elapsed time where reported, and from the source authors’ own stage descriptor where it is not. (Additive; does not alter registered scope.)

R1.2 (further justification for an RM-orthosis-specific review). The rationale for an RMO-specific review is generally convincing. However, given that several recent qualitative and patient-experience studies have emerged in this area, the authors may wish to further highlight how the growing patient-perspective evidence base has created the need for a dedicated synthesis. This may help further distinguish the current review from previous RMO reviews that primarily focused on clinical outcomes and orthosis application.

Response.

We have strengthened this rationale, drawing on Section A. The revised “Existing evidence syntheses” paragraph — shown in full in the REVISED TEXT block under R2 detailed — include Brosnan in the existing-syntheses paragraph, below — now notes the emergence of dedicated qualitative RM-orthosis research since 2016 and frames the present review as the patient-experience-indexed synthesis this growing but dispersed evidence now warrants — complementary to, and building on, Hirth et al. and Brosnan et al.

Change made: Introduction — addressed within the revised “Existing evidence syntheses” paragraph, which now names Brosnan et al. and the emergence of qualitative RM research. No separate REVISED TEXT block is shown here, to avoid reproducing the same paragraph twice; it appears in full at the block named in the response above.

R1.3 (transparency of coding across domains). The conceptual framework of acceptability, usability, and everyday impact is a notable strength of the protocol. However, several patient-reported constructs (e.g., comfort, satisfaction, wearability, and adherence-related experiences) may reasonably overlap across domains. Although the protocol indicates that multi-domain coding will be used when appropriate, providing additional coding examples or decision rules within the extraction framework may further improve transparency and reproducibility. This may be particularly useful when different reviewers are required to classify findings that could plausibly fit more than one conceptual category.

Response.

We agree. The protocol already specifies multi-domain coding (lines 349–352). We have added anchor coding guidance and an explicit inter-reviewer decision rule to the Data analysis and presentation section, and fuller anchor examples — including verbatim coding examples — to the extraction instrument (S3 Appendix).

To be added to the Data analysis and presentation section, following the multi-domain-coding provision (line 352) and the stage-of-use sentence added under R1.1:

REVISED TEXT

To support consistent classification, comfort is coded under acceptability where it reflects the patient’s evaluative appraisal of the orthosis and under usability where it reflects practical wearability during tasks, with multi-domain coding applied where a single finding spans both; satisfaction, wearability, and adherence-related experiences are coded analogously against the operational definitions in the inclusion criteria. Where two reviewers classify a finding differently, the finding is coded to each applicable domain and the discrepancy resolved by discussion, or by a third reviewer where needed.

Change made: Data analysis and presentation — added the coding rule above. S3 Appendix — added fuller anchor examples, with verbatim coding examples, and the same decision rule. (Additive; strengthens the existing multi-domain-coding provision.)

C. Response to Reviewer #2

R2 General (a). The

Attachment Submitted filename: Response_to_Reviewers.docx

# Peer review: Decision Letter 1

**DOI:** 10.1371/journal.pone.0349852.r003

**Authors:** Hiroki Annaka

22 Aug 2026

Dear Dr. Chockalingam,

Thank you for submitting your manuscript to PLOS One. After careful consideration, we feel that it has merit but does not fully meet PLOS One’s publication criteria as it currently stands. Therefore, we invite you to submit a revised version of the manuscript that addresses the points raised during the review process.

==============================

Please consider making revisions based on the reviewer's comments.

==============================

Please submit your revised manuscript by Oct 06 2026 11:59PM. If you will need more time than this to complete your revisions, please reply to this message or contact the journal office at plosone@plos.org. When you're ready to submit your revision, log on to https://www.editorialmanager.com/pone/ and select the 'Submissions Needing Revision' folder to locate your manuscript file.

- A letter that responds to each point raised by the academic editor and reviewer(s). You should upload this letter as a separate file labeled 'Response to Reviewers'.
- A marked-up copy of your manuscript that highlights changes made to the original version. You should upload this as a separate file labeled 'Revised Manuscript with Track Changes'.
- An unmarked version of your revised paper without tracked changes. You should upload this as a separate file labeled 'Manuscript'.

If you would like to make changes to your financial disclosure, please include your updated statement in your cover letter. Guidelines for resubmitting your figure files are available below the reviewer comments at the end of this letter.

If applicable, we recommend that you deposit your laboratory protocols in protocols.io to enhance the reproducibility of your results. Protocols.io assigns your protocol its own identifier (DOI) so that it can be cited independently in the future. For instructions see: https://journals.plos.org/plosone/s/submission-guidelines#loc-laboratory-protocols. Additionally, PLOS One offers an option for publishing peer-reviewed Lab Protocol articles, which describe protocols hosted on protocols.io. Read more information on sharing protocols at https://plos.org/protocols?utm_medium=editorial-email&utm_source=authorletters&utm_campaign=protocols.

As the corresponding author, your ORCID iD is verified in the submission system and will appear in the published article. PLOS supports the use of ORCID, and we encourage all coauthors to register for an ORCID iD and use it as well. Please encourage your coauthors to verify their ORCID iD within the submission system before final acceptance, as unverified ORCID iDs will not appear in the published article. *Only*  the individual author can complete the verification step; PLOS staff *cannot*  verify ORCID iDs on behalf of authors.

We look forward to receiving your revised manuscript.

Kind regards,

Hiroki Annaka

Academic Editor

PLOS One

Journal Requirements:

1. If the reviewer comments include a recommendation to cite specific previously published works, please review and evaluate these publications to determine whether they are relevant and should be cited. There is no requirement to cite these works unless the editor has indicated otherwise.

2. Please review your reference list to ensure that it is complete and correct. If you have cited papers that have been retracted, please include the rationale for doing so in the manuscript text, or remove these references and replace them with relevant current references. Any changes to the reference list should be mentioned in the rebuttal letter that accompanies your revised manuscript. If you need to cite a retracted article, indicate the article’s retracted status in the References list and also include a citation and full reference for the retraction notice.

[Note: HTML markup is below. Please do not edit.]

Reviewer's Responses to Questions

**Comments to the Author**

1. Does the manuscript provide a valid rationale for the proposed study, with clearly identified and justified research questions?

Reviewer #1: Yes

Reviewer #2: Yes

**********

2. Is the protocol technically sound and planned in a manner that will lead to a meaningful outcome and allow testing the stated hypotheses?

Reviewer #1: Yes

Reviewer #2: Yes

**********

3. Is the methodology feasible and described in sufficient detail to allow the work to be replicable?

Reviewer #1: Yes

Reviewer #2: Yes

**********

4. Have the authors described where all data underlying the findings will be made available when the study is complete?

The PLOS Data policy

Reviewer #1: Yes

Reviewer #2: Yes

**********

5. Is the manuscript presented in an intelligible fashion and written in standard English?

Reviewer #1: Yes

Reviewer #2: Yes

**********

Please use the space provided to explain your answers to the questions above and, if applicable, provide comments about issues authors must address before this protocol can be accepted for publication. You may also include additional comments for the author, including concerns about research or publication ethics.

You may also provide optional suggestions and comments to authors that they might find helpful in planning their study.

Reviewer #1: Thank you to the authors for the careful and comprehensive revision of the manuscript. I have reviewed the revised protocol and the responses to my previous comments.

The authors have adequately addressed all three points raised in my initial review. In particular, the revised manuscript now clarifies how patient-perspective findings will be considered according to the stage of orthosis use, strengthens the rationale for a dedicated RM-orthosis patient-perspective synthesis in light of Brosnan et al. (2026), and improves the transparency and reproducibility of the conceptual coding framework.

I also appreciate the authors’ careful engagement with the recently published review by Brosnan et al. (2026), which more clearly positions the proposed review as complementary to, rather than duplicative of, the existing evidence base.

Overall, my previous concerns have been satisfactorily addressed, and I have no further substantive comments.

I recommend acceptance of the revised manuscript.

Reviewer #2: Dear authors,

Thank you for addressing the feedback previously provided.

A final request is to please update lines 51 and 52 "...repairs, the terms RME-plus and RMF-plus denote the addition of a wrist component and RME-only and RMF-only its absence, following Brosnan et al. [2]." to have the plus and only in italics as per Brosnan et al. Please also put the only and plus in italics throughout your manuscript.

Once complete, I support the study protocol for publication. I wish you well in undertaking the scoping review and look forward to reading it once published. Kind Regards

**********

what does this mean?). If published, this will include your full peer review and any attached files.

If you choose “no”, your identity will remain anonymous but your review may still be made public.

**Do you want your identity to be public for this peer review?**  For information about this choice, including consent withdrawal, please see our Privacy Policy

Reviewer #1: No

Reviewer #2: No

**********

[NOTE: If reviewer comments were submitted as an attachment file, they will be attached to this email and accessible via the submission site. Please log into your account, locate the manuscript record, and check for the action link "View Attachments". If this link does not appear, there are no attachment files.]

To ensure your figures meet our technical requirements, please review our figure guidelines: https://journals.plos.org/plosone/s/figures

You may also use PLOS’s free figure tool, NAAS, to help you prepare publication quality figures: https://journals.plos.org/plosone/s/figures#loc-tools-for-figure-preparation.

NAAS will assess whether your figures meet our technical requirements by comparing each figure against our figure specifications.

# Author comment: Author response to Decision Letter 2

**DOI:** 10.1371/journal.pone.0349852.r004

22 Aug 2026

Dear Dr Annaka and Reviewers,

Thank you for your assessment of the revised protocol. We are grateful to Reviewer 1 for recommending acceptance, and to Reviewer 2 for supporting the protocol for publication.

Reviewer 2 made one final request: to italicise the suffixes "plus" and "only" in the terms RME-plus, RMF-plus, RME-only, and RMF-only, following the styling used by Brosnan et al. We have done this. These terms appear in the manuscript only in the defining sentence at lines 51-52, where the styling has now been applied; we have applied the same styling to the identical terms in the S3 data-extraction appendix, so that the convention is consistent across the submission. No wording was changed; this is a typographical correction only.

No other changes have been made, as no further revisions were requested.

Thank you again for your time and for supporting this protocol.

Kind regards,

Manigandan Chockalingam, on behalf of all authors

Attachment Submitted filename: Response_to_Reviewers_auresp_2.docx

# Editor report: Decision Letter 2

**DOI:** 10.1371/journal.pone.0349852.r005

**Authors:** Hiroki Annaka

24 Aug 2026

Patient-perspective evidence on the acceptability, usability, and everyday impact of relative motion orthoses in adult hand rehabilitation: A scoping review protocol

PONE-D-26-20165R2

Dear Dr. Chockalingam,

We’re pleased to inform you that your manuscript has been judged scientifically suitable for publication and will be formally accepted for publication once it meets all outstanding technical requirements.

Within one week, you’ll receive an e-mail detailing the required amendments. When these have been addressed, you’ll receive a formal acceptance letter and your manuscript will be scheduled for publication.

An invoice will be generated when your article is formally accepted. Please note, if your institution has a publishing partnership with PLOS and your article meets the relevant criteria, all or part of your publication costs will be covered. Please make sure your user information is up-to-date by logging into Editorial Manager at Editorial Manager® and clicking the ‘Update My Information' link at the top of the page. For questions related to billing, please contact billing support.

If your institution or institutions have a press office, please notify them about your upcoming paper to help maximize its impact. If they’ll be preparing press materials, please inform our press team as soon as possible -- no later than 48 hours after receiving the formal acceptance. Your manuscript will remain under strict press embargo until 2 pm Eastern Time on the date of publication. For more information, please contact onepress@plos.org.

Kind regards,

Hiroki Annaka

Academic Editor

PLOS One

Additional Editor Comments (optional):

Reviewers' comments:

# Editor report: Acceptance letter

**DOI:** 10.1371/journal.pone.0349852.r006

**Authors:** Hiroki Annaka

PONE-D-26-20165R2

PLOS One

Dear Dr. Chockalingam,

I'm pleased to inform you that your manuscript has been deemed suitable for publication in PLOS One. Congratulations! Your manuscript is now being handed over to our production team.

At this stage, our production department will prepare your paper for publication. This includes ensuring the following:

* All references, tables, and figures are properly cited

* All relevant supporting information is included in the manuscript submission,

* There are no issues that prevent the paper from being properly typeset

You will receive further instructions from the production team, including instructions on how to review your proof when it is ready. Please keep in mind that we are working through a large volume of accepted articles, so please give us a few days to review your paper and let you know the next and final steps.

Lastly, if your institution or institutions have a press office, please let them know about your upcoming paper now to help maximize its impact. If they'll be preparing press materials, please inform our press team within the next 48 hours. Your manuscript will remain under strict press embargo until 2 pm Eastern Time on the date of publication. For more information, please contact onepress@plos.org.

You will receive an invoice from PLOS for your publication fee after your manuscript has reached the completed accept phase. If you receive an email requesting payment before acceptance or for any other service, this may be a phishing scheme. Learn how to identify phishing emails and protect your accounts at https://explore.plos.org/phishing.

If we can help with anything else, please email us at customercare@plos.org.

Thank you for submitting your work to PLOS One and supporting open access.

Kind regards,

PLOS One Editorial Office Staff

on behalf of

Dr. Hiroki Annaka

Academic Editor

PLOS One
